# DDX39B drives colorectal cancer progression by promoting the stability and nuclear translocation of PKM2

**DOI:** 10.1038/s41392-022-01096-7

**Published:** 2022-08-17

**Authors:** Gang Zhao, Hang Yuan, Qin Li, Jie Zhang, Yafei Guo, Tianyu Feng, Rui Gu, Deqiong Ou, Siqi Li, Kai Li, Ping Lin

**Affiliations:** Lab of Experimental Oncology, State Key Laboratory of Biotherapy and Cancer Center, and Frontiers Science Center for Disease-Related Molecular Network, West China Hospital, Sichuan University, Chengdu, Sichuan 610041 China

**Keywords:** Gastrointestinal cancer, Metastasis

## Abstract

Metastasis is a major cause of colorectal cancer (CRC) mortality, but its molecular mechanisms are still not fully understood. Here, we show that upregulated DDX39B correlates with liver metastases and aggressive phenotypes in CRC. DDX39B is an independent prognostic factor associated with poor clinical outcome in CRC patients. We demonstrate that Sp1 potently activates DDX39B transcription by directly binding to the GC box of the DDX39B promoter in CRC cells. DDX39B overexpression augments the proliferation, migration, and invasion of CRC cells, while the opposite results are obtained in DDX39B-deficient CRC cells. Mechanistically, DDX39B interacts directly with and stabilizes PKM2 by competitively suppressing STUB1-mediated PKM2 ubiquitination and degradation. Importantly, DDX39B recruits importin α5 to accelerate the nuclear translocation of PKM2 independent of ERK1/2-mediated phosphorylation of PKM2, leading to the transactivation of oncogenes and glycolysis-related genes. Consequently, DDX39B enhances glucose uptake and lactate production to activate Warburg effect in CRC. We identify that Arg319 of DDX39B is required for PKM2 binding as well as PKM2 nuclear accumulation and for DDX39B to promote CRC growth and metastasis. In addition, blocking PKM2 nuclear translocation or treatment with glycolytic inhibitor 2-deoxy-D-glucose efficiently abolishes DDX39B-triggered malignant development in CRC. Taken together, our findings uncover a key role for DDX39B in modulating glycolytic reprogramming and aggressive progression, and implicate DDX39B as a potential therapeutic target in CRC.

## Introduction

Colorectal cancer (CRC) represents the third most common human malignancy and the second leading cause of cancer-related deaths. Globally, more than 1.8 million new cases of CRC are diagnosed annually, and the incidence of CRC at younger ages (diagnosed age less than 50 years) is increasing rapidly, especially in developed countries.^1^ There are several risk factors including germline APC and MLH1 gene mutations, obesity, poor diets, and physical inactivity that increase CRC incidence.^2^ Approximately 60% of new CRC cases are diagnosed at an advanced stage, which leads to high mortality.^3^ Despite recent advances in the treatment of CRC, the outcomes of these patients remain poor due to metastasis and recurrence.^4–6^ Accumulating evidence has shown that CRC cells possess a high level of abnormal gene expression or mutational burden and extensive molecular heterogeneity.^7–9^ Thus, further elucidation of the underlying molecular mechanisms accounting for the progressive and aggressive characteristics of CRC cells will contribute to the development of biomarker-driven therapeutic targets for individual CRC patients.

Emerging studies have shown that metabolic reprogramming is essential for tumorigenesis and metastasis. Cancer cells depend on elevated rates of glucose uptake and lactate production in the presence of abundant oxygen supply, concomitant with a reduced rate of oxidative phosphorylation. This condition is known as aerobic glycolysis or the Warburg effect, a hallmark of cancer.^10,11^ Pyruvate kinase (PK) is a pivotal rate-limiting enzyme that mediates the final step of glycolysis, catalyzing the production of pyruvate, and simultaneously phosphorylating ADP to produce ATP. Four PK isoforms (M1, M2, L, and R) have been identified in mammals. The L (PKL) and R (PKR) isoforms are found in liver cells and red blood cells, respectively.^12^ Alternate pre-mRNA splicing of the M isoform (PKM) produces PKM1 containing exon 9 of the PKM gene, which is expressed in most adult tissues, and PKM2 containing exon 10, which is mainly present in fetal tissues and cancer cells.^13^ This alternative splicing process is modulated by three heterogeneous nuclear ribonucleoproteins (hnRNPs), hnRNPI, hnRNPA1, and hnRNPA2, leading to PKM2 mRNA production.^14^ It has been shown that fructose 1,6-bisphosphate (FBP) can trigger a reversible dimer-to-tetramer conversion of PKM2 to allosterically activate PKM2, while PKM1 presents as homotetramer with a highly constitutive activity that is independent of FBP.^15^ Upregulated PKM2 has been observed in diverse human cancers, and the switch from PKM1 to PKM2 is critical for cancer cell survival.^16,17^ In addition to its canonical PK role in glycolysis, PKM2 can be translocated into the nucleus, where it functions as a protein kinase and a cotranscription factor, resulting in transactivation of metabolic and proliferative genes, including c-Myc, GLUT1, and MEK5.^18–20^ However, the molecular mechanisms underlying the dynamic accumulation and nuclear localization of PKM2 during tumorigenesis are still not fully known.

Human DExD-box helicase 39B (DDX39B), which is a member of the DEAD-box protein family, has been implicated in many steps of RNA metabolism, including pre-mRNA splicing, transcription, mRNA export, ribosome biogenesis, translation initiation, and mRNA degradation.^21,22^ A previous study showed that DDX39B prevented and removed the accumulation of RNA–DNA hybrids in the active chromatin that maintained genome stability and subsequently assisted transcription and replication.^23^ Depletion of DDX39B, as well as its Drosophila homolog Hel25E, resulted in nuclear accumulation of long circRNAs.^24^ Research into the role of DDX39B in tumorigenesis has recently expanded. For example, DDX39B upregulation caused progressive telomere elongation and increased cell viability in telomerase-positive cancer cells, suggesting an important role for DDX39B in telomere maintenance and genome integrity.^25^ DDX39B interacted with Luzp4, enhancing its function as an mRNA export adapter, which promoted the growth of melanoma cells in vitro.^26^ With the increase in “omics” studies, abnormal transcripts and changes in protein levels of DDX39B were found in breast cancer and melanoma.^27,28^ However, the functional role and detailed mechanism of DDX39B in glucose metabolism reprogramming that contributes to the initiation and development of CRC remains unclear.

In the present study, we analyze two GEO datasets to screen the dysregulated genes involved in CRC aggressive progression and identify that DDX39B is gradually increased from normal mucosa to primary tumor to metastatic CRC tissues. Enforced DDX39B expression enhances the proliferation and metastasis of CRC cells both in vitro and in vivo. Mechanistically, our data show that DDX39B prevents the ubiquitination and degradation of PKM2 through competitive interaction with STUB1 and subsequently recruits importin α5 to facilitate the nuclear translocation of PKM2, which ultimately activates the aerobic glycolysis of CRC cells. We also demonstrate that Arg319 of DDX39B is essential for PKM2 binding and the oncogenic function of DDX39B in CRC. Taken together, our study highlights the significance of DDX39B in the liver metastases and clinical outcome of CRC patients and reveal a novel mechanism by which DDX39B accelerates the nuclear translocation of PKM2 independent on ERK1/2-mediated phosphorylation of PKM2.

## Results

### Upregulation of DDX39B correlates with aggressive phenotypes and poor prognosis in human CRCs

**Fig. 1:**
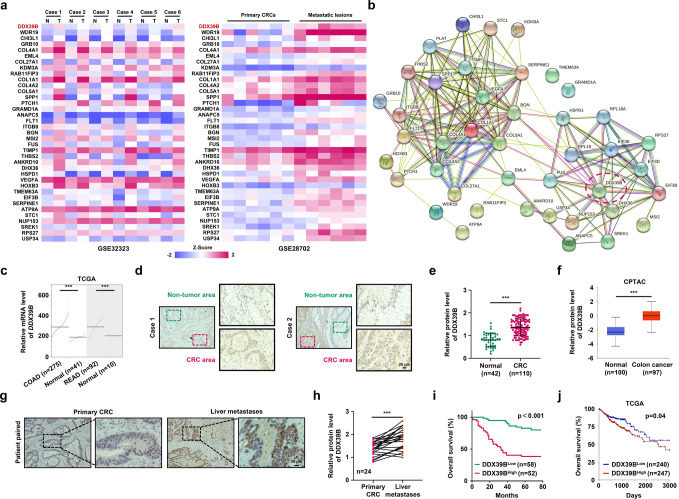
Upregulation of DDX39B correlates with aggressive phenotypes and poor prognosis in human CRC. A total of 37 up-regulated genes both in primary and metastatic CRC tumors were displayed as a heatmap (**a**) and by STRING network (**b**) analysis. N normal tissues; T: CRC tumors. **c** DDX39B transcripts in colon adenocarcinoma (COAD), rectal adenocarcinoma (READ) and normal tissues from the TCGA database were analyzed through an online tool (http://gepia2.cancer-pku.cn/). **d**, **e** DDX39B protein levels in a microarray of CRC and normal mucosal tissues were detected by immunohistochemistry (IHC) staining. **d** Representative images are shown, and **e** the relative staining intensities were analyzed by two-tailed unpaired *t* test. **f** DDX39B protein level in normal and colon cancer tissues from the Clinical Proteomic Tumor Analysis Consortium (CPTAC) database was analyzed through an online tool (http://ualcan.path.uab.edu/index.html). **g**, **h** DDX39B protein expression in primary CRCs and patient-paired liver metastases were detected by IHC. **g** Representative images are shown, and **h** the relative staining intensities were analyzed by two-tailed paired *t* test. **i** The correlation between DDX39B protein level and the overall survival of CRC patients was tested by Kaplan-Meier analysis. **j** Kaplan-Meier survival analysis for DDX39B transcripts in colon adenocarcinoma patients was carried out using an online tool (https://xenabrowser.net/). ***p < 0.001

To identify molecules that are potentially involved in the initiation and progression of CRC, we investigated GEO datasets for gene expression differences between CRC samples and paired adjacent noncancerous tissues (GSE32323), and between primary and metastatic lesions from CRC patients (GSE28702). The overlapping analysis revealed a total of 37 genes that were gradually increased from normal mucosa to primary tumor to metastatic CRC (Fig. 1a). STRING network analysis indicated that these upregulated genes could be mainly classified into two subgroups (Fig. 1b). Because DDX39B is in the center of one subgroup and its functional role in CRC is unclear, we chose DDX39B for further investigation. From the TCGA database, significant increases in DDX39B mRNA were observed in colon adenocarcinoma and rectal adenocarcinoma (READ) tissues compared with normal tissues (Fig. 1c). Moreover, we used the TCGA database to analyze the correlation of DDX39B with other genes in the same subgroup and found that DDX39B expression was positively associated with DHX36, EIF3B, EIF3D, EIF3E, FUS, HSPD1, NUP153, RPL18A, RPL19, RPS27, SREK1, and USP34 in colon adenocarcinoma (Supplementary Fig. 1a). Similar results were observed in READ, except for RPL18A (Supplementary Fig. 1b). We next assessed the expression of DDX39B protein in our tissue microarray containing 110 samples of CRCs and 42 samples of normal mucosal tissues. Immunohistochemical analysis demonstrated that higher protein levels of DDX39B were detected in CRC tissues than in adjacent nontumor tissues (Fig. 1d, e). Consistent with our results, upregulated DDX39B protein expression was observed in the Clinical Proteomic Tumor Analysis Consortium colon cancer database (Fig. 1f). Importantly, we found that DDX39B was further augmented in liver metastases from CRC compared with patient-paired primary tumors (Fig. 1g, h). We also evaluated the expression of DDX39B in primary and metastatic tumor tissues from melanoma, breast cancer, and prostate cancer patients. Compared with primary lesions, DDX39B was augmented in metastatic prostate cancer tissues, but not in melanoma and breast cancer, suggesting that DDX39B may serve as a potential predictor of metastasis risk for certain cancers, including CRC and prostate cancer (Supplementary Fig. 2). Elevated DDX39B protein expression was correlated with larger tumor size, poor histological grade, increased tumor invasion, higher incidence of metastasis to both regional lymph nodes and distant organs, and advanced AJCC stage (Supplementary Table 1). Notably, CRC patients with high DDX39B expression had worse outcomes than those with low DDX39B expression (Fig. 1i). A consistent result was obtained from the TCGA database (Fig. 1j). Multivariate analysis showed that DDX39B was an independent prognostic factor associated with poor overall survival in CRC patients (Supplementary Table 2). These data indicate a positive correlation of DDX39B expression with aggressive progression and poor prognosis of CRCs.

### Sp1 activates DDX39B transcription in CRC cells

**Fig. 2:**
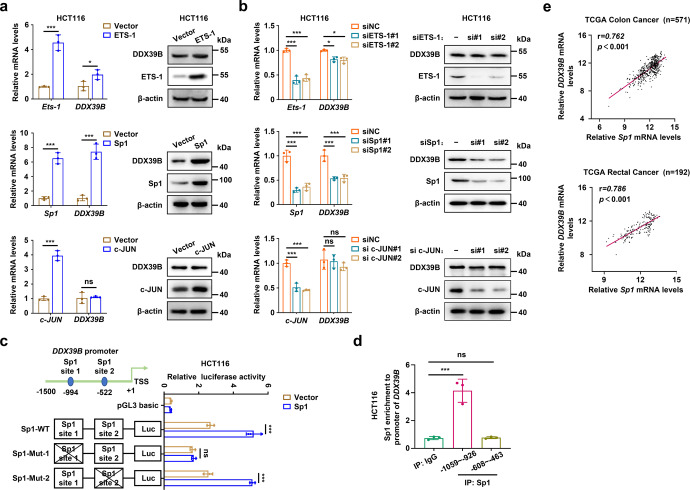
Sp1 activates DDX39B transcription in CRC cells. **a** HCT116 cells were transiently transfected with the plasmids of ETS-1, Sp1, or c-JUN, respectively. The mRNA and protein levels of DDX39B were measured by qPCR and western blotting. **b** HCT116 cells were transiently transfected with siRNAs targeting ETS-1, Sp1, or c-JUN, respectively. The mRNA and protein expressions of DDX39B were determined by qRT-PCR and western blotting. **c** The relative luciferase activity was detected in HCT116 cells transfected with the indicated DDX39B promoter in the presence or absence of Sp1. **d** DNA fragments from HCT116 cells were immunoprecipitated with the Sp1-specific antibody and analyzed by qPCR using the indicated primers. **e** The correlations of transcript levels between DDX39B and Sp1 in human colon and rectal adenocarcinoma tissues were analyzed through an online tool (https://xenabrowser.net/). Data are presented as mean ± SD. The *p* values were obtained by two-tailed unpaired *t* test (**a**) or one-way ANOVA (**b–d**). *p < 0.05, ***p < 0.001, ns not significant

To identify the mechanism of upregulated DDX39B in CRC, we first analyzed the promoter of DDX39B and found putative binding sites of several transcription factors, including Ets-1, Sp1, and c-Jun. As shown in Fig. 2a and Supplementary Fig. 3a, exogenous introduction of Sp1 markedly enhanced the mRNA and protein levels of DDX39B, while a moderate increase in DDX39B expression was observed in CRC cells with enforced Ets-1. Knockdown of Sp1 or Ets-1 resulted in a strong or modest decline in DDX39B levels, respectively (Fig. 2b and Supplementary Fig. 3b). However, c-Jun was unable to induce DDX39B expression (Fig. 2a, b and Supplementary Fig. 3a, b). Thus, we selected Sp1 for further validation. There were two conserved Sp1 binding sites in the DDX39B promoter, and we generated a luciferase reporter plasmid containing the wild-type or Sp1 binding site mutant DDX39B promoter (Fig. 2c). A luciferase reporter assay showed that Sp1 site 1 mutant, but not Sp1 site 2 mutant, diminished the basal luciferase activity compared with the wild-type DDX39B promoter (Fig. 2c and Supplementary Fig. 3c). We observed higher luciferase activity of the wild-type DDX39B promoter in the presence of Sp1, whereas this effect was largely prevented in the Sp1 binding site 1 mutant DDX39B promoter (Fig. 2c and Supplementary Fig. 3c). Chromatin immunoprecipitation assays demonstrated that endogenous Sp1 protein was able to bind to the DNA fragment (−1059/−926) of the DDX39B promoter in HCT116 and SW620 cells (Fig. 2d and Supplementary Fig. 3d). In addition, Sp1 expression was positively associated with DDX39B in colon and rectal adenocarcinoma patients from the TCGA database (Fig. 2e). These results reveal that Sp1 induces the upregulation of DDX39B transcription by directly binding to the DDX39B promoter in CRC.

### DDX39B promotes CRC growth and metastasis in vitro and in vivo

**Fig. 3:**
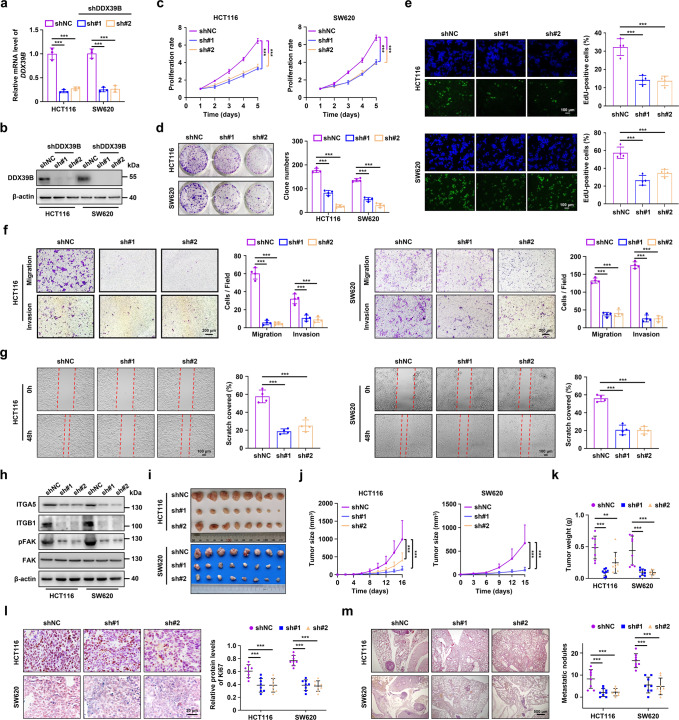
DDX39B knockdown inhibits CRC growth and metastasis in vitro and in vivo. Knockdown efficiency mediated by two lentivirus-delivered DDX39B-specific shRNA (sh#1 and sh#2) was verified by qRT-PCR (**a**) and western blotting (**b**) in HCT116 and SW620 cells. The cell viability and proliferation of DDX39B-knockdown CRC cells were measured by CCK8 assays (**c**), colony formation assays (**d**), and EdU assays (**e**). The motility of DDX39B-knockdown CRC cells was examined by transwell migration/invasion assays (**f**) and wound healing assays (**g**). **h** The protein levels of ITGA5, ITGB1, pFAK^Y397^, and FAK in DDX39B-knockdown CRC cells were detected by western blotting. The growth and metastatic abilities of DDX39B-knockdown CRC cells in vivo were assessed in nude mice by subcutaneous and lung metastasis tumor models (*n* = 8), respectively. The images (**i**), tumor sizes (**j**), tumor weights (**k**), and Ki-67 expression (**l**) of subcutaneous xenografts are presented. Representative pulmonary metastases detected by H&E staining are shown, along with the number of metastatic nodules (**m**). Data are presented as mean ± SD. The *p* values were obtained by two-way ANOVA (**c, j**) or one-way ANOVA (others). **p < 0.01, ***p < 0.001

To explore the functional role of DDX39B in CRC, we first examined the expression of DDX39B protein in five CRC cell lines. Our results showed that the DDX39B protein levels were augmented in highly metastatic cell lines (LoVo and SW620) compared with weakly metastatic cell lines (HT29, HCT116, and SW480) (Supplementary Fig. 4). Subsequently, we modulated DDX39B expression with two DDX39B-specific short hairpin RNAs (shDDX39B#1 and shDDX39B#2) and DDX39B^Flag^ cDNA using lentivirus-mediated delivery into HCT116 and SW620 cells, respectively. The shRNA-mediated knockdown of DDX39B and cDNA-mediated DDX39B overexpression in SW620 and HCT116 cells were confirmed by qPCR and western blotting (Fig. 3a, b and Supplementary Fig. 5a, b). DDX39B silencing reduced the proliferation of SW620 and HCT116 cells, as determined by CCK-8, anchorage-dependent colony formation and EdU assays (Fig. 3c–e). In contrast, DDX39B overexpression significantly promoted the growth of CRC cells (Supplementary Fig. 5c–e). Transwell assays showed that DDX39B suppression decreased the migration and invasion of SW620 and HCT116 cells (Fig. 3f), whereas exogenous introduction of DDX39B enhanced the motility of CRC cells (Supplementary Fig. 5f). A similar result was observed in a wound-healing assay (Fig. 3g and Supplementary Fig. 5g). We subsequently evaluated the expression of components of the integrin/FAK signaling pathway (including ITGA5, ITGB1, and pFAK^Y397^), as they are critical effectors in promoting the metastasis of cancer cells.^29,30^ We found that DDX39B knockdown resulted in decreased protein levels of ITGA5, ITGB1, and pFAK^Y397^ (Fig. 3h). The opposite result was obtained in DDX39B-overexpressing CRC cells (Supplementary Fig. 5h).

To further determine whether DDX39B was involved in the progression of CRC in vivo, we first examined the function of DDX39B on tumor growth by generating subcutaneous tumor models in nude mice. The size and weight of xenograft tumors in the DDX39B-deficient groups were significantly delayed compared with those in the negative control group (Fig. 3i–k), while the tumors derived from the DDX39B overexpression group were larger and heavier than those in the mock group (Supplementary Fig. 5i–k). A similar result was observed with the Ki-67 staining assay (Fig. 3l and Supplementary Fig. 5l). We next evaluated the role of DDX39B in CRC metastasis in vivo using a lung metastasis model via tail-vein injection. DDX39B deficiency resulted in fewer lung metastatic nodules (Fig. 3m), whereas DDX39B overexpression accelerated CRC lung metastasis (Supplementary Fig. 5m). Taken together, these data confirm that DDX39B promotes CRC growth and metastasis in vitro and in vivo.

### DDX39B interacts with PKM2 and prevents its degradation

**Fig. 4:**
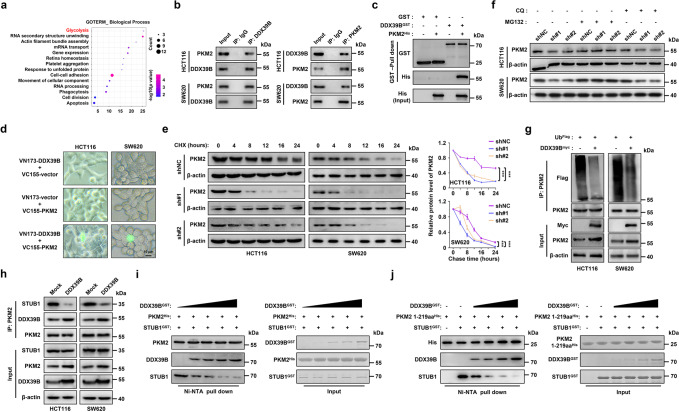
DDX39B interacts with PKM2 and reduces its degradation. **a** The Gene Ontology analysis of DDX39B interactome. **b** The endogenous interaction between DDX39B and PKM2 was examined by immunoprecipitation assay in CRC cells. **c** GST pull-down assays were carried out with bacterially expressed GST-fused DDX39B and His-fused PKM2. **d** HCT116 and SW620 cells were transfected with pBiFC-VN173-DDX39B and/or pBiFC-VC155-PKM2, and the fluorescence signals were imaged. **e** The degradation of PKM2 protein in DDX39B knockdown CRC cells was measured by cycloheximide (CHX) chase analysis. **f** DDX39B-deficient CRC cells were treated with MG132 or chloroquine (CQ), and PKM2 protein levels were determined by western blotting. **g** The indicated CRC cells were transfected with plasmids expressing Ub^Flag^ and DDX39B^myc^, and cell lysates were immunoprecipitated with anti-PKM2 antibody followed by western blotting. **h** The binding affinity of PKM2 and STUB1 in CRC cells stably expressing mock or DDX39B was measured by immunoprecipitation assay. In vitro competitive binding analysis was executed with the indicated purified proteins, and the effect of DDX39B on PKM2^WT^-STUB1 (**i**) or PKM2^1–218,219*aa*^-STUB1 (**j**) binding was determined by western blotting. Data represent as mean ± SD. The *p* values were determined by two-way ANOVA (**e**). ***p < 0.001

To elucidate the molecular mechanism of DDX39B-mediated CRC progression, we performed immunoaffinity purification and mass spectrometry (MS) analysis of DDX39B interactors. A number of proteins coimmunoprecipitated with DDX39B (Supplementary Table 3), including DDX39A, ALYREF, SARNP, U2AF2, THOC1, and components of the TRanscription-EXport complex or spliceosome, which was in agreement with previous studies.^31–33^ Gene Ontology analysis revealed that the highest-enrichment biological process of the DDX39B interactome was glycolysis, which contained GAPDH, PFKP, and PKM (Fig. 4a). We observed that endogenous DDX39B coimmunoprecipitated with PKM2, GAPDH, and PFKP in CRC cells (Fig. 4b and Supplementary Fig. 6a). PFKP and PKM2 are rate-limiting metabolic enzymes in the glycolytic process, while PKM2 has been reported to play key roles in regulating glycolytic flux and carcinogenesis of CRC. Therefore, we focused on PKM2 for further investigation. A GST pull-down assay showed that DDX39B could directly bind to PKM2 in vitro (Fig. 4c). To further explore the binding of DDX39B with PKM2 in living CRC cells, we performed a bimolecular fluorescence complementation assay. The nonfluorescent fragments (VN173 and VC155) derived from a yellow fluorescent protein (Venus) were fused with DDX39B and PKM2, respectively, and the expression levels of VN173-DDX39B and VC155-PKM2 were validated by western blotting (Supplementary Fig. 6b). The reconstituted fluorophores from the DDX39B and PKM2 complexes were visualized in whole cells (Fig. 4d). Similar results were revealed by immunoprecipitation and immunofluorescence assays (Supplementary Fig. 6c, d), suggesting that DDX39B interacted with PKM2 in both the cytoplasm and the nucleus. To determine which domain of PKM2 interacted with DDX39B, we generated four HA-tagged truncations of PKM2 as described in a previous study.^34^ The 1-219 aa and 1-390 aa truncations as well as the full-length PKM2 retained the ability to bind to DDX39B (Supplementary Fig. 6e), suggesting that the N-terminal region of PKM2 (1-219 aa) was responsible for its binding to DDX39B. Because PKM1 and PKM2 share identical N-terminal amino acid sequences (including amino acids 1-219), we examined whether DDX39B interacts with PKM1 and found an association of DDX39B with PKM1 under physiological conditions (Supplementary Fig. 6f). We next evaluated whether DDX39B could regulate the expression of PKM1/2 in CRC cells. In DDX39B-overexpressing cells, there was no significant change in the mRNA levels of PKM1 and PKM2 (Supplementary Fig. 6g). However, upregulation of DDX39B increased the protein level of PKM2 but not PKM1 (Supplementary Fig. 6h), suggesting that DDX39B may contribute to the protein stability of PKM2 but not PKM1. Consistent with this result, cycloheximide chase analysis demonstrated that increased DDX39B expression significantly enhanced the protein stability of PKM2 but not PKM1 (Supplementary Fig. 6i, j). In contrast, silencing DDX39B accelerated PKM2 protein degradation (Fig. 4e).

To further investigate the molecular pathway involved in DDX39B-modulated PKM2 degradation, DDX39B-deficient cells were treated with MG132 (a proteasome inhibitor) or chloroquine (CQ, a lysosome inhibitor). Our data showed that knockdown of DDX39B decreased PKM2 protein levels in the presence of CQ, while this effect was abolished by MG132 exposure (Fig. 4f). These results indicated that DDX39B stabilized PKM2 by repressing ubiquitin–proteasome pathway-mediated PKM2 degradation. Consistent with this observation, DDX39B overexpression significantly reduced the ubiquitination of PKM2 (Fig. 4g). Since a previous study reported that STUB1 (STIP1 homology and U-box-containing protein 1) functioned as an E3 ligase to facilitate the ubiquitination and degradation of PKM2,^35^ we tested the effect of DDX39B on STUB1 expression. Interestingly, the mRNA and protein levels of STUB1 were not altered by DDX39B (Supplementary Fig. 6k, l). However, DDX39B overexpression weakened the binding affinity between PKM2 and STUB1 (Fig. 4h). Given the direct interaction between DDX39B and PKM2, we next carried out a competition analysis to estimate the effect of DDX39B on PKM2-STUB1 binding. The results demonstrated that DDX39B competed with STUB1 for PKM2 binding in a dose-dependent manner (Fig. 4i). As mentioned above, PKM2 1-219 aa was required for the association with DDX39B, and we further clarified whether PKM2 1-219 aa contributed to its interaction with STUB1. As shown in Fig. 4j, the purified recombinant PKM2 1-219 aa protein could directly bind to STUB1. However, this combination was gradually repressed by the increased amount of purified DDX39B protein (Fig. 4j). Taken together, these data indicate that DDX39B directly binds to PKM2 and enhances its stability by competitively suppressing STUB1-mediated PKM2 ubiquitination and degradation.

### DDX39B promotes ERK-independent PKM2 nuclear translocation

**Fig. 5:**
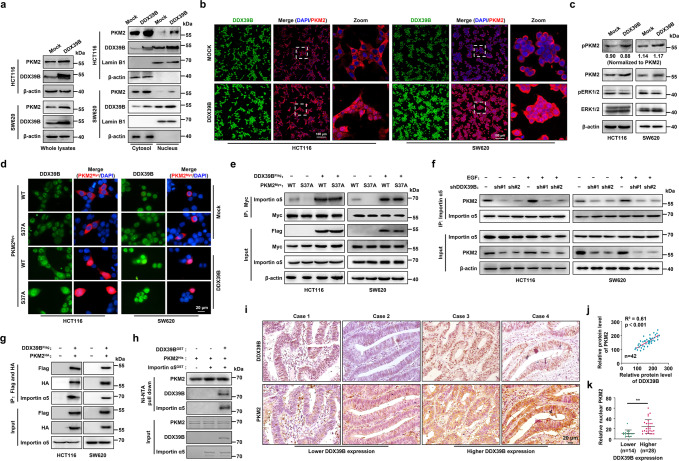
DDX39B promotes ERK-independent PKM2 nuclear translocation. **a** Nuclear and cytosolic protein lysates prepared from HCT116 and SW620 cells stably expressing mock or DDX39B were assayed by western blotting. **b** The subcellular localization of PKM2 in HCT116 and SW620 cells stably expressing mock or DDX39B was visualized by immunofluorescence assay. **c** Phosphorylation of PKM2^S37^ and ERK1^T202/Y204^, ERK2^T185/Y187^ in CRC cells stably expressing mock or DDX39B was detected by western blotting. **d** The indicated CRC cells were cotransfected with Flag-DDX39B and Myc-PKM2^WT^ or Myc-PKM2^S37A^, and immunofluorescence assay was performed. **e** The CRC cells were cotransfected as indicated, and the binding of importin α5 to PKM2^WT^ or PKM2^S37A^ was evaluated by immunoprecipitation assay. **f** DDX39B-knockdown CRC cells were treated with or without 150 ng/ml EGF, and the association of importin α5 with PKM2 was detected by immunoprecipitation assay. **g** Lysates prepared from CRC cells cotransfected with Flag-DDX39B and HA-PKM2 were subjected to tandem affinity purification using anti-Flag and anti-HA magnetic beads. **h** In vitro binding analysis was performed using the indicated purified proteins. **i** DDX39B and PKM2 protein levels in serial sections of CRC tissues (*n* = 42) were measured by immunohistochemistry staining. **j** The correlation of relative staining intensities between DDX39B and PKM2. **k** The association of DDX39B and nuclear PKM2 in CRC tissues. The *p* values were determined by Pearson correlation coefficient analysis (**j**) or Student’s *t* test (**k**). **p < 0.01

Because our preceding data provided evidence that DDX39B elevated PKM2 protein levels, we next investigated whether DDX39B regulated the PK activity of PKM2 in CRC cells. The results showed that DDX39B did not alter the total intracellular PK activity in CRC cells (Supplementary Fig. 7a). Meanwhile, the PK activity of recombinant PKM2 (rPKM2) protein was not affected by recombinant DDX39B (rDDX39B) in vitro (Supplementary Fig. 7b). Interestingly, we found that DDX39B overexpression depressed the tetramer but concomitantly increased the monomeric and dimeric forms of endogenous PKM2 in HCT116 cells (Supplementary Fig. 7c). Thus, we inferred that the elevated level of PKM2 caused by DDX39B introduction did not alter the total intracellular PK activity in CRC cells, which might be attributed to the increased amount of inactive monomer and less active dimer of PKM2. As a previous study revealed that ERK1/2 phosphorylates PKM2 at Ser37 and recruits importin α5 to promote PKM2 translocation into the nucleus upon EGFR activation,^36^ we next determined whether DDX39B was involved in the phosphorylation and nuclear translocation of PKM2. Fractionation analysis showed that DDX39B overexpression increased the nuclear PKM2 level (Fig. 5a). A similar result was exhibited by the immunofluorescence assay (Fig. 5b). Conversely, knockdown of DDX39B diminished the expression and nuclear accumulation of PKM2 (Supplementary Fig. 7d). Western blotting analysis showed that DDX39B overexpression increased PKM2^S37^ phosphorylation, whereas DDX39B deprivation eliminated PKM2 S37 phosphorylation (Fig. 5c and Supplementary Fig. 7e). However, DDX39B did not affect the ratio of PKM2^S37^ phosphorylation to total PKM2 or ERK1/2 phosphorylation, suggesting that the elevated level of PKM2^S37^ phosphorylation caused by DDX39B may be attributed to the increase in total PKM2 protein expression (Fig. 5c and Supplementary Fig. 7e). Notably, DDX39B enhanced the nuclear distribution of PKM2 in the presence of the MAP-kinase kinase (MEK) inhibitor U0126 (Supplementary Fig. 7f), suggesting that DDX39B-induced PKM2 nuclear translocation may be independent of the ERK1/2-mediated phosphorylation of PKM2 at S37. In agreement with this result, we showed that Myc-PKM2^WT^ and Myc-PKM2^S37A^ were distributed in the cytoplasm without DDX39B introduction, while DDX39B promoted the nuclear translocation of both PKM2^WT^ and the PKM2^S37A^ mutant (Fig. 5d). Moreover, DDX39B induced a comparable binding affinity between PKM2^WT^ or PKM2^S37A^ and importin α5 (Fig. 5e). In contrast, DDX39B deprivation abrogated EGF-induced nuclear accumulation of PKM2 as well as the interaction between PKM2 and importin α5 (Fig. 5f and Supplementary Fig. 7g). Tandem affinity purification analysis showed that DDX39B, PKM2, and importin α5 existed in the form of a complex in vivo (Fig. 5g). To further illustrate the impact of DDX39B on the association between PKM2 and importin α5, we employed the recombinant purified proteins DDX39B, PKM2, and importin α5 for the in vitro binding assay. Our results showed that PKM2 alone did not interact with importin α5. Notably, PKM2 is bound to importin α5 in the presence of DDX39B, suggesting that DDX39B may recruit importin α5 to promote PKM2 translocation (Fig. 5h). We also found a positive correlation between DDX39B levels and total PKM2 protein expression in CRC samples and xenograft tissues (Fig. 5i, j and Supplementary Fig. 7h, i). In addition, CRC cells with higher DDX39B expression tended to have elevated nuclear PKM2 in CRC sections (Fig. 5k and Supplementary Fig. 7j, k). Together, these results indicate that DDX39B promotes the nuclear translocation of PKM2 independent of ERK1/2-mediated phosphorylation of PKM2.

### DDX39B promotes aerobic glycolysis by enhancing nuclear PKM2 function in CRC cells

**Fig. 6:**
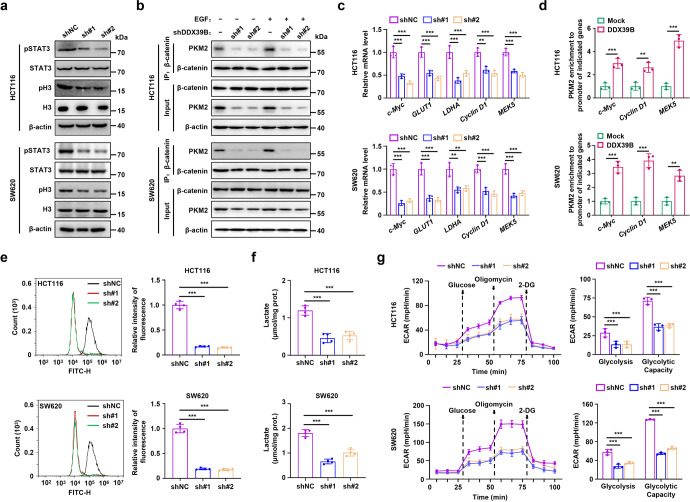
DDX39B enhances nuclear PKM2 function and aerobic glycolysis in CRC cells. **a** Phosphorylation of STAT3^Y705^ and histone H3^T11^ in DDX39B-knockdown CRC cells was detected by western blotting. **b** DDX39B-deficient CRC cells were treated with or without 150 ng/ml EGF, and the association of PKM2 with β-catenin was detected by immunoprecipitation assay. **c** The relative mRNA levels of c-Myc, GLUT1, LDHA, Cyclin D1, and MEK5 were measured by qPCR in CRC cells stably expressing shNC or shDDX39B. **d** Binding of PKM2 to the promoters of c-Myc, Cyclin D1, and MEK5 was detected by chromatin immunoprecipitation analysis. **e** Glucose uptake in DDX39B-knockdown CRC cells was detected by flow cytometry using the fluorescent glucose analog 2-NBDG. **f** The lactate levels in DDX39B-deficient CRC cells were quantified. **g** The extracellular acidification rate (ECAR) of DDX39B-knockdown CRC cells was monitored, and the levels of glycolysis and glycolytic capacity were calculated. Data are presented as mean ± SD. The *p* values were determined by Student’s *t* test (**d**) or one-way ANOVA (others). **p < 0.01, ***p < 0.001

As PKM2 has been reported to function as a protein kinase and cotranscription factor with β-catenin in the nucleus, we first explored the impact of DDX39B on the phosphorylation of histone H3 and STAT3, which are two known substrates of PKM2. Our results showed that the levels of phosphorylated STAT3^Y705^ and histone H3^T11^ were significantly decreased upon DDX39B deficiency, while DDX39B overexpression increased the phosphorylation of STAT3^Y705^ and histone H3^T11^ (Fig. 6a and Supplementary Fig. 8a). We next examined whether DDX39B could affect the binding of PKM2 to β-catenin. As shown in Supplementary Fig. 8b, DDX39B facilitated the binding of PKM2 to β-catenin. Conversely, DDX39B suppression blocked the EGF-induced association between PKM2 and β-catenin (Fig. 6b). Notably, we observed no direct interaction between PKM2 and β-catenin in the presence or absence of DDX39B (Supplementary Fig. 8c). These findings indicated that the DDX39B-mediated enhanced interaction between PKM2 and β-catenin was attributed to the increased nuclear accumulation of PKM2. To further assess the functional consequences of DDX39B-mediated nuclear translocation of PKM2, we measured the transcript levels of PKM2 target genes. PKM2-dependent β-catenin transactivation enhanced the expression of the cyclin D1 and c-Myc genes, which promoted the transcription of glucose transporter 1 (GLUT1) and lactate dehydrogenase A (LDHA).^37^ PKM2 activates the transcription of MEK5 by phosphorylating STAT3 at Y705.^19^ Our data revealed that DDX39B silencing blunted, whereas DDX39B overexpression elevated, the mRNA expression levels of the c-Myc, GLUT1, LDHA, Cyclin D1, and MEK5 genes (Fig. 6c and Supplementary Fig. 8d). Chromatin immunoprecipitation analysis showed that ectopic expression of DDX39B augmented PKM2 recruitment to the promoters of the c-Myc, Cyclin D1, and MEK5 genes (Fig. 6d). Subsequently, we determined whether DDX39B could affect glycolytic flux in CRC cells. As shown in Fig. 6e, knockdown of DDX39B attenuated uptake of the glucose analog 2-NBDG into SW620 and HCT116 cells. The opposite result was obtained in CRC cells overexpressing DDX39B (Supplementary Fig. 8e). DDX39B-deficient CRC cells exhibited significant reductions in intracellular lactate levels and the extracellular acidification rate (ECAR) compared with the negative control group (Fig. 6f, g). In contrast, upregulated DDX39B strengthened lactate production as well as the basal glycolysis and glycolytic capacity of CRC cells (Supplementary Fig. 8f, g). Taken together, these results indicate that DDX39B enhances the function of nuclear PKM2, leading to the transactivation of oncogenes and glycolytic genes that trigger the Warburg effect in CRC cells.

### Arg319 of DDX39B is required for PKM2 binding and for the ability of DDX39B to promote carcinogenesis and development in CRC

**Fig. 7:**
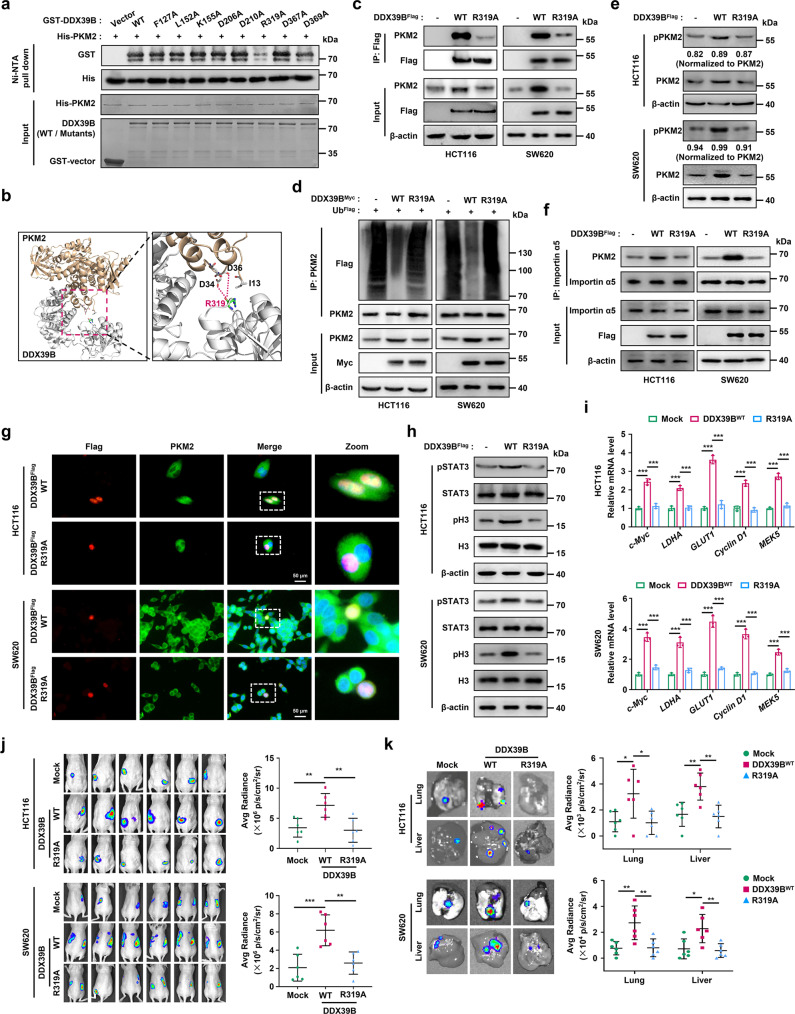
Arg319 of DDX39B is required for PKM2 binding and the promotion of CRC carcinogenesis and metastasis. **a** A Ni-NTA pull-down assay was carried out using bacterially-expressed wild type and the indicated mutant forms of GST-tagged DDX39B and His-tagged PKM2 in vitro. **b** The binding interface between DDX39B and PKM2 was based on the molecular docking model. **c–f**, **h–k** The indicated CRC cells were divided into three groups: stably expressing mock, DDX39B^WT^, and DDX39B^R319A^. **c** Interaction between PKM2 with DDX39B^WT^ or DDX39B^R319A^ was determined by immunoprecipitation assay. **d** Cell lysates were immunoprecipitated with anti-PKM2 antibody, and the ubiquitination of PKM2 was detected. **e** The phosphorylation of PKM2^S37^ was determined by western blotting. **f** Binding of PKM2 with importin α5 was examined by immunoprecipitation assay. **g** HCT116 and SW620 cells were transfected with the indicated plasmids, and subcellular localization of DDX39B and PKM2 signals were observed by immunofluorescence assay. **h** The phosphorylation of STAT3^Y705^ and histone H3^T11^ were detected by western blotting. (**i**) The relative transcription of c-Myc, GLUT1, LDHA, Cyclin D1 and MEK5 genes was measured by qPCR. The indicated CRC cells were orthotopically inoculated into the cecum of mice (*n* = 6). At day 60 after inoculation, the bioluminescent images and light emission of orthotopic tumors were captured and quantified (**j**). The representative bioluminescent images of the isolated lungs and livers were obtained, and the metastases were quantified (**k**). Data are presented as mean ± SD. The *p* values were obtained by one-way ANOVA. *p < 0.05, **p < 0.01, ***p < 0.001

We next sought to identify the potential sites of DDX39B required for PKM2 binding. Utilizing the available crystal structures of DDX39B (PDB ID: 1XTI) and PKM2 (PDB ID: 1T5A) in the Research Collaboratory for Structural Bioinformatics protein databank, we carried out a protein–protein docking test (http://zdock.umassmed.edu/) and chose the top conformation for molecular dynamics simulation (Supplementary Fig. 9a). The binding free energy and the energy component for the DDX39B-PKM2 complex were calculated (Supplementary Table 4 and Supplementary Fig. 9b) and the eight amino acids of DDX39B that principally contributed to its association with PKM2 were selected for further investigation. The Ni-NTA pull-down assay showed that the R319A mutation in DDX39B, but not other mutations, significantly eliminated the direct interaction between DDX39B and PKM2 (Fig. 7a). Based on our modeling of the molecular docking of DDX39B with PKM2, the positively charged arginine residue (R319) of DDX39B was located at the binding interface, and two negatively charged aspartic acid residues (D34 and D36) of PKM2 were located near R319 (Fig. 7b). Subsequently, we explored the functional role of the mutated protein DDX39B^R319A^ in CRC cells. For this purpose, we generated CRC cell lines stably expressing DDX39B^WT^ or DDX39B^R319A^ cDNA. As shown in Fig. 7c, the mutated DDX39B^R319A^ dramatically decreased PKM2 expression and binding affinity to PKM2 in vivo compared with DDX39B^WT^. DDX39B^R319A^ also failed to repress the ubiquitination and degradation of PKM2 or to augment PKM2^S37^ phosphorylation and the binding affinity of PKM2 with importin α5 (Fig. 7d–f). Moreover, the DDX39B^R319A^ mutant was unable to promote PKM2 nuclear translocation, the phosphorylation of STAT3^Y705^ and H3^T11^, or the transactivation of PKM2 target genes (Fig. 7g–i). Functionally, DDX39B^R319A^ did not promote cell viability, colony formation, migration, metastasis-related marker expression, glucose uptake or intracellular lactate production compared with wild type DDX39B (Supplementary Fig. 9c–h). Consistent with the in vitro findings, the orthotopic colorectal cancer model showed that the carcinogenic and metastatic abilities in vivo were greatly decreased in CRC cells expressing DDX39B^R319A^ compared with CRC cells expressing DDX39B^WT^ (Fig. 7j, k). Together, these data indicate that Arg319 of DDX39B is required for PKM2 binding and for DDX39B to promote initiation and progression in CRC.

### The nuclear PKM2-mediated Warburg effect is indispensable for DDX39B-triggered tumorigenicity and metastasis in CRC

**Fig. 8:**
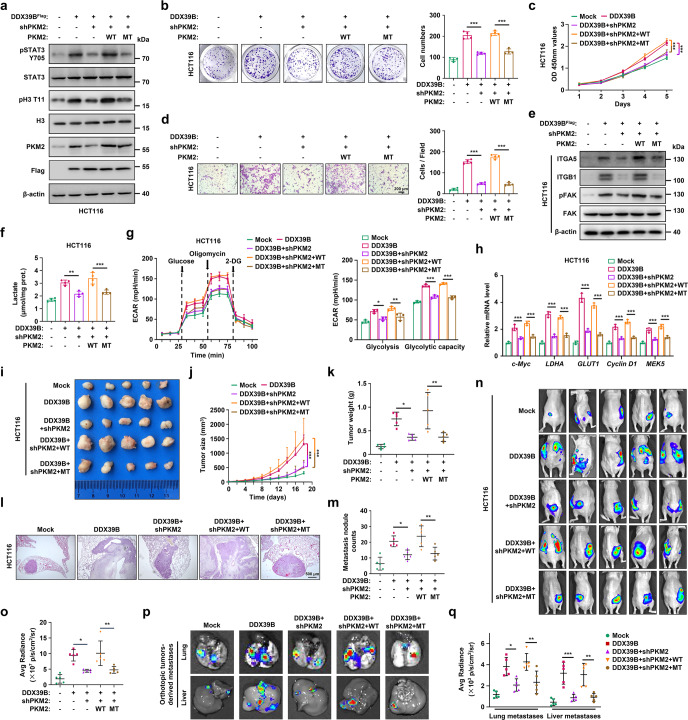
Blocking PKM2 nuclear accumulation impairs DDX39B-triggered Warburg effect and tumorigenicity in CRC. **a–q** HCT116 cells simultaneously expressing DDX39B cDNA and PKM2-shRNA were re-introduced into RNAi-resistant PKM2^WT^ or PKM2^R399/400A^ mutant (MT), respectively. **a** Phosphorylation of STAT3^Y705^ and histone H3^T11^ was detected by western blotting. **b** The cell proliferation was measured by colony formation assay. **c** Cell viability was measured by CCK8 assay. **d** Cell motility was determined by transwell migration assays. **e** The indicated protein levels were detected by western blotting. **f** The lactate production in indicated HCT116 cells was quantified. **g** The extracellular acidification rate (ECAR) of indicated HCT116 cells was monitored, and the levels of glycolysis and glycolytic capacity were calculated. **h** The relative transcriptions of c-Myc, GLUT1, LDHA, Cyclin D1 and MEK5 were measured by qPCR. The growth and metastatic abilities of indicated HCT116 cells in vivo were assessed in nude mice by subcutaneous and lung metastasis tumor models (*n* = 5), respectively. The images (**i**), tumor sizes (**j**), and tumor weights (**k**) of subcutaneous xenografts are presented. Representative pulmonary metastases detected by H&E staining are shown (**l**), along with the number of metastatic nodules (**m**). **n–q** Indicated HCT116 cells were orthotopically inoculated into the cecum of mice (*n* = 5). At day 60 after inoculation, the bioluminescent images of orthotopic tumors were captured (**n**) and light emissions were quantified (**o**). The representative bioluminescent images of the isolated lungs and livers were obtained (**p**), and the metastases were quantified (**q**). Data are presented as mean ± SD. The *p* values were obtained by two-way ANOVA (**c, j**) or one-way ANOVA (others). *p < 0.05, **p < 0.01, ***p < 0.001

As our above results demonstrated that DDX39B promoted the nuclear translocation of PKM2, we next explored whether the tumorigenicity and metastasis induced by DDX39B in CRC depended on PKM2 nuclear translocation. The R399/400A mutant, which was previously demonstrated to arrest the interaction between PKM2 and importin α5, blocked PKM2 nuclear translocation.^36^ Our data showed that DDX39B facilitated the nuclear translocation of PKM2^WT^ but not PKM2^R399/400A^ (Supplementary Fig. 10a, b). Subsequently, we tested the effect of the PKM2^R399/400A^ mutant on DDX39B-mediated carcinogenesis. We generated CRC cell lines (HCT116 and SW620) stably expressing DDX39B cDNA and PKM2-shRNA together and reintroduced shRNA-resistant PKM2^WT^ or PKM2^R399/400A^, respectively (Fig. 8a and Supplementary Fig. 11a). Western blotting assays showed that the elevation of STAT3^Y705^ and H3^T11^ phosphorylation caused by DDX39B was abrogated by PKM2 blockade (Fig. 8a and Supplementary Fig. 11a). Notably, CRC cells expressing wild-type PKM2 exhibited higher levels of phosphorylated STAT3 and H3 than those expressing the R399/400A mutant PKM2 (Fig. 8a and Supplementary Fig. 11a). Moreover, knockdown of PKM2 abolished DDX39B-enhanced proliferation, migratory ability, and metastasis-related marker expression in HCT116 and SW620 cells, which were rescued by reintroduction of PKM2^WT^ but not PKM2^R399/400A^ (Fig. 8b–e and Supplementary Fig. 11b–e). Similar results were observed with respect to lactate production, glycolytic flux and PKM2 target gene transcription (Fig. 8f–h and Supplementary Fig. 11f–h). The subcutaneous xenograft and pulmonary metastasis tumor models consistently showed that CRC cells expressing DDX39B along with PKM2^WT^ developed larger tumors and more lung metastatic nodules than cells expressing DDX39B along with PKM2^R399/400A^ (Fig. 8i–m and Supplementary Fig. 12a–e). Last, we created an orthotopic colorectal cancer model and confirmed that cells expressing DDX39B along with PKM2^WT^ accelerated the growth of CRC in situ and increased CRC-derived metastases compared with cells expressing DDX39B along with PKM2^R399/400A^ (Fig. 8n–q and Supplementary Fig. 12f–i). To further clarify the impact of aerobic glycolysis on DDX39B-triggered CRC development, DDX39B-overexpressing HCT116 cells were treated with the glycolytic inhibitor 2-deoxy-D-glucose (2-DG). DDX39B overexpression enhanced the colony formation, cell viability, migration, and lactate level of CRC cells, which were reversed by 2-DG addition (Supplementary Fig. 13a–d). We also found that 2-DG treatment significantly antagonized the increased tumor growth and metastasis formation in vivo caused by DDX39B upregulation (Supplementary Fig. 13e–i). Collectively, our results indicate that nuclear PKM2-mediated glycolytic-dominant metabolic reprogramming is crucial for DDX39B-stimulated tumorigenesis and metastasis in CRC.

## Discussion

Metastasis is a complicated, multistep process and the major cause of CRC-associated mortality. Clarifying the molecular mechanism of CRC metastasis is critical to evaluate the risk of its occurrence and formulate corresponding therapeutic strategies for CRC patients. In the present study, we identified 37 upregulated genes in metastatic CRC compared with primary tumors and normal mucosa. Increased expression of VEGFA has been observed in several cancers, including CRC, and bevacizumab (anti-VEGF monoclonal antibody) plus fluorouracil-based combination chemotherapy significantly improved the response rate, progression-free survival and overall survival in patients with metastatic CRC.^38^ Secreted phosphoprotein 1 overexpression promotes the metastasis of CRC through activation of epithelial–mesenchymal transition.^39^ TIMP1 protein, a natural inhibitor of matrix metalloproteinases, is upregulated in advanced CRC and positively associated with lymphatic invasion and distant metastasis of CRC.^40,41^ In addition, collagen family members, including COL1A1 and COL4A1, as ligands of integrins modulate cell adhesion and invasion that contribute to the malignant progression of CRC.^42,43^ Notably, our present study provided the first evidence that DDX39B was elevated in liver metastatic lesions compared with patient-paired primary CRC tumors. Higher DDX39B expression was positively associated with aggressive phenotypes and poor outcome in patients with CRC. We identified Sp1 as a potent transcriptional activator of DDX39B, and the mutation of GC box (known as the Sp1 binding motif) binding site 1 in the DDX39B promoter considerably interrupted the enhanced luciferase activity caused by Sp1. We also found a positive correlation between Sp1 and DDX39B in colon and rectal adenocarcinoma patients from the TCGA database. Consistent with our findings, Sp1 has been shown to augment the malignant development of CRC through transcriptional activation of long noncoding RNAs.^44,45^ Our study discovered a novel downstream effector of Sp1 in CRC. Moreover, we demonstrated that DDX39B promoted CRC growth and metastasis by activating PKM2-mediated glycolytic reprogramming. When this work was in progress, another group recently reported that DDX39B facilitated the proliferation and metastasis of CRC through enhancement of CDK6/CCND1 and FUT3 pre-mRNA splicing, respectively.^46,47^ These studies focused on the canonical RNA helicase DDX39B, while our work uncovered an important connection between DDX39B and glucose metabolism alterations and expanded the nonhelicase function of DDX39B via protein–protein interactions. Unlike tumors such as estrogen receptor-positive breast cancer which takes years or decades to develop into metastatic entities, systemic dissemination and colonization to regional lymph nodes or distant tissues can rapidly occur during progression from colorectal adenomas to locally invasive carcinomas.^48,49^ Given the roles of DDX39B in the invasion and metastasis of CRC, we propose that the evaluation of DDX39B expression may provide a potential predictor of metastasis risk for individual CRC patients.

The upregulation of PKM2 or the replacement of PKM1 by PKM2, which triggers aerobic glycolysis, is known to occur in various cancers.^50^ Unlike PKM1, which retains a higher constitutive PK activity, the low activity of PKM2 allows cancer cells to accumulate diverse glycolytic intermediates required for macromolecule biosynthesis of nucleic acids, amino acids, and phospholipids, and boosts anabolism through the pentose-phosphate pathway.^51–53^ Moreover, accumulated PKM2 in the cytoplasm can translocate into the nucleus and function as a protein kinase and transcriptional coactivator to regulate the transcription of oncogenic and metabolic genes, leading to rapid cell proliferation and tumorigenesis. In the present study, we reported that DDX39B directly interacted with the N-terminal region of PKM2 and increased the protein level of PKM2 but not the mRNA level. We also found that DDX39B enhanced the stability of the PKM2 protein. Interestingly, although DDX39B could bind to PKM1, it had no effect on the expression and stability of PKM1 protein. Our data suggest a unique role of DDX39B in the regulation of the PKM2 protein. Recent reports have demonstrated that PKM2 can be degraded by the ubiquitin-proteasome pathway or chaperone-mediated autophagy.^35,54^ Here, our data showed that the proteasome inhibitor MG132, but not the lysosome inhibitor CQ, effectively prevented the degradation of the PKM2 protein in the absence of DDX39B. In addition, we further demonstrated that DDX39B competed with the E3 ligase STUB1 for PKM2 binding, subsequently blocking PKM2 ubiquitination and degradation, and ultimately leading to PKM2 protein accumulation. We also identified that PKM2 expression was positively correlated with DDX39B levels in CRC samples and xenograft tissues. Thus, our study reveals a novel mechanism for the upregulation of PKM2 protein by DDX39B.

A previous study showed that EGFR activation promoted ERK1/2-dependent phosphorylation of PKM2 at Ser37 and enhanced the association of PKM2 with the nuclear import protein importin α5 to facilitate the nuclear translocation of PKM2.^36^ Herein, we demonstrated that DDX39B augmented the nuclear distribution of PKM2 in the presence of the ERK activation inhibitor U0126 as well as the nuclear translocation of a PKM2 phosphorylation-deficient mutant (S37A). This mechanism of DDX39B-mediated nuclear translocation of PKM2 may provide an explanation for the treatment failure of selumetinib (a MEK inhibitor in phase II and III clinical trials that blocks the activation of ERK1/2) in clinical application, and DDX39B may serve as an available biomarker for patient selection and response rate assessment in clinical treatment of MEK inhibitors. The molecular docking model showed that the positively charged arginine residue (R319) of DDX39B interacted with the two negatively charged aspartic acid residues of PKM2 (D34 and D36), which are located near S37. This may explain why DDX39B promoted PKM2 nuclear accumulation independent of ERK1/2-mediated phosphorylation of PKM2 at S37. Consistently, the DDX39B R319A mutant failed to trigger PKM2 nuclear translocation and inhibited the carcinogenic ability of DDX39B in CRC. Moreover, we found consistent expression patterns of DDX39B and nuclear PKM2 protein in CRC samples and xenograft tissues. Importantly, DDX39B augmented the Warburg effect by nuclear PKM2-dependent transcription, supporting the expression of GLUT1 and LDHA and leading to increased glucose uptake and lactate production and eventually the initiation and development of CRC, as a constitutively cytosolic PKM2 mutant (R399/400A) reversed the DDX39B-mediated enhanced proliferation and migratory ability of CRC cells. Since DDX39B did not alter the PK activity of PKM2, our data provide evidence supporting the model that DDX39B contributes to the nonmetabolic activity of PKM2 by promoting the nuclear translocation of PKM2.

**Fig. 9:**
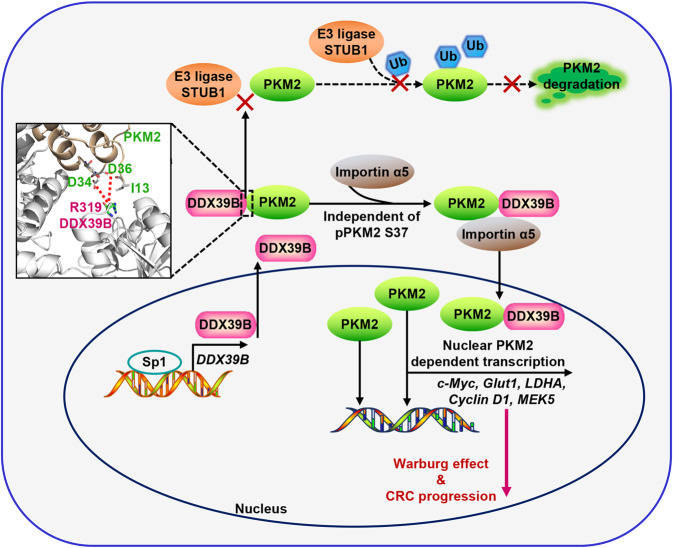
Schematic diagram of the Sp1/DDX39B/PKM2 axis in regulation of the Warburg effect and CRC progression

In summary, we established DDX39B as a key promoter of the Warburg effect that contributes to the malignant progression of CRC. Sp1 potently activates DDX39B transcription by directly binding to the GC box of the DDX39B promoter in CRC cells. DDX39B physically interacts with PKM2, which depresses the degradation of PKM2 caused by STUB1-mediated ubiquitination and subsequently accelerates the nuclear translocation of PKM2 in an ERK1/2-mediated phosphorylation-independent manner, where nuclear PKM2 functions as a protein kinase and transcriptional coactivator to regulate the expression of oncogenic and metabolic genes, leading to cell proliferation and metastasis (Fig. 9). Our findings reveal the significant clinical relevance and prognostic value of DDX39B and provide key mechanistic insights into the DDX39B-PKM2 axis triggering inherent metabolic plasticity in CRC. Future screening of inhibitors targeting DDX39B or blocking DDX39B-PKM2 binding may provide a promising therapeutic approach for CRC treatment.

## Materials and methods

### Animal experiments

All animal experiments were approved by the Institutional Animal Care and Use Committee (IACUC) of West China Hospital Sichuan University. 4-to-6 week-old female athymic nude mice (BALB/cJGpt-Foxn1^nu^/Gpt) were obtained from Gempharmatech Co., Ltd., Jiangsu, China. All mice were fed standard rodent forage and maintained under specific-pathogen-free (SPF) conditions at an ambient temperature of 23 °C and 12 h/12 h light/dark cycle. For the subcutaneous tumor model, the indicated CRC cells (2 × 10^6^ cells in 100 μL PBS per mouse) were subcutaneously injected into the dorsal flanks of nude mice. The longest diameter (*A*) and shortest diameter (*B*) of each xenograft were measured every 2 days, and the tumor volumes (calculated as 0.5 × *A* × *B*^2^) were monitored within a month after inoculation. At the end of the experiment the mice were euthanized by narcotic overdose and the tumors were removed, weighed, and used for later testing. For the lung metastasis model, the indicated CRC cells (1 × 10^6^ cells in 50 μL PBS per mouse) were injected via the tail vein. Two months after inoculation, the mice were euthanized, their lungs were removed, paraffin embedded and serial sections were prepared. Sections were H&E stained and metastatic nodules were counted. In the orthotopic CRC model, mice were anesthetized and the indicated CRC cells stably expressing luciferase (2 × 10^6^ cells suspended in 50% PBS and 50% Matrigel per mouse) were injected into the serosa of the cecum. Two months after inoculation, mice were intraperitoneally injected with D-luciferin (150 mg/kg per mouse), and the bioluminescent images and light emission of primary tumors, and metastases among isolated lungs and livers were captured and quantified with an IVIS Spectrum in vivo imaging system.

### Human tumor tissues

Clinical specimens in this study (cohort 1), including normal colorectal tissues (*n* = 42) and CRC tissues (*n* = 110) from previously untreated colorectal carcinoma patients undergoing surgical resection from 2008 and 2009 were obtained from West China Biobanks, Department of Clinical Research Management, West China Hospital, Sichuan University. Cohort 2 (*n* = 24) was collected from patients who underwent surgical resection for colorectal cancer liver metastasis with matched primary tumor tissue available from 2010 to 2013. The median expression of DDX39B was used to define subgroups. Informed consent was obtained from each patient prior to any specimen-related studies and this study was approved by the Ethics Committee on Biomedical Research, West China Hospital of Sichuan University.

### GST or Ni-NTA pull-down assay

GST- and His- fused recombinant proteins were expressed in *E.coli* BL21 (DE3) and purified with glutathione Sepharose (GE Healthcare) or Ni-NTA agarose (Invitrogen), respectively, according to the manufacturer’s protocol. Recombinant proteins were incubated with glutathione Sepharose beads or Ni-NTA agarose beads overnight at 4 °C, then beads were washed five times with 0.1% NP-40 buffer and bound protein was eluted for immunoblotting analysis.

### Glucose uptake analysis

The indicated cells (5 × 10^5^ cells per well) were seeded into 24-well plates and cultured for 24 h. Cells were then starved for 4 h in glucose-free medium and a fluorescent glucose analog (100 μM 2-NBDG, Sigma) was added to the medium. After incubation for 2 h, cellular fluorescence was quantitated by flow cytometry (LSRFortessa, Becton Dickinson).

### Co-immunoprecipitation followed by mass spectrometry (MS)

Lysates prepared from CRC cells stably expressing FLAG-vector or FLAG-DDX39B were immunoprecipitated with anti-FLAG M2 magnetic beads. The protein complexes captured by the beads were separated by SDS-PAGE and visualized using Femto SDS-PAGE staining buffer (Affinibody Life Science, AG) for 10 min. The differential gel bands of interest and their corresponding negative bands were removed from the gel and cut into small pieces. The gel bands were dehydrated with acetonitrile, reduced with 5 mM 1, 4-dithiothreitol, alkylated with 11 mM iodoacetamide, and digested overnight in trypsin. The peptides were purified by ZipTip (Millipore), and the extracted mixtures were resuspended in 0.1% formic acid +2% acetonitrile and analyzed by LC-MS/MS using an Orbitrap Exploris mass spectrometer (Thermo-Fisher). The obtained RAW files were analyzed by the Proteome Discoverer Software (v2.2), and proteins were identified by comparison against the Homo_sapiens_9606_SP_20171214.fasta database (20,239 sequences). The corresponding reverse database matching against the data was used to calculate the false discovery rate (FDR) of peptide using the following search parameters: digestion method, trypsin (full); allowed missing cleavages, 2; minimum peptide length, 6 amino acid residues; precursor peptide mass tolerance, 10 ppm; fragment ion mass tolerance, 0.02 Da; and maximum FDR of peptides, 1%.

### Pyruvate kinase (PK) activity assay

The PK activity of cell extracts or rPKM2 was measured using a PK activity colorimetric assay kit (Biovision) according to the users’ manual. Extracts prepared from the indicated cells or purified rPKM2 (200 ng) combined with or without rDDX39B (200 ng), were incubated with reaction buffer at 25 °C for 20 min. PK activity was calculated as the decreasing rate of OD at 570 nm, and normalized to protein concentration.

### Glycolysis stress test

The Seahorse XFe 24 Analyzer (Agilent) was used to monitor the ECAR of CRC cells according to the operating manual. Briefly, the CRC cells were incubated in pyruvate- and glucose-free assay medium containing 2 mM glutamine at pH 7.4 for 1 h at 37 °C, in a non-CO_2_ incubator. After incubation, 10 mM glucose, 1 μM oligomycin, and 50 mM 2-dexoy-D-glucose, were added to each well. The data for basic glycolysis and glycolytic capacity levels of cells were analyzed using Wave 2.3 software.

### Statistical analyses

All experiments in vitro were done independently with at least three biological replicates. The data presentation form and statistical analyses are given in the corresponding figure legends. Data analyses were carried out with GraphPad Prism (v8.0, La Jolla, USA). *p* values <0.05 were considered as statistically significant.

## Supplementary information

Supplementary Materials file

Supplemental Figure 1

Supplemental Figure 2

Supplemental Figure 3

Supplemental Figure 4

Supplemental Figure 5

Supplemental Figure 6

Supplemental Figure 7

Supplemental Figure 8

Supplemental Figure 9

Supplemental Figure 10

Supplemental Figure 11

Supplemental Figure 12

Supplemental Figure 13

Supplemental Table 1

Supplemental Table 2

Supplemental Table 3

Supplemental Table 4

Supplemental Table 5

Supplemental Table 6

## Data Availability

All data are available within the main article, supplementary materials, or available from the corresponding author upon reasonable request.
