# Supplementary material for: DDX39B drives colorectal cancer progression by promoting the stability and nuclear translocation of PKM2
Source: Signal Transduct Target Ther. 2022 Aug 17;7:275. doi: 10.1038/s41392-022-01096-7 (PMC9381590; doi:10.1038/s41392-022-01096-7)
Supplement: Supplementary file 1 — Supplementary Materials file [file 41392_2022_1096_MOESM1_ESM.docx]

Supplementary Materials for

**DDX39B drives colorectal cancer progression by promoting the stability and nuclear translocation of PKM2**

Gang Zhao^1,2^, Hang Yuan^1,2^, Qin Li^1^, Jie Zhang^1^, Yafei Guo^1^, Tianyu Feng^1^, Rui Gu^1^, Deqiong Ou^1^, Siqi Li^1^, Kai Li^1,*^ and Ping Lin^1,*^

Correspondence to: linping@scu.edu.cn; likai@wchscu.cn.

**This PDF file includes:**

Materials and Methods

Supplementary Figures. S1 to S13

**Other Supplementary Materials for this manuscript include the following:**

Supplementary Tables. S1 to S6

Supplementary Table S1. Correlation between DDX39B expression and clinicopathological characteristics of CRC patients.

Supplementary Table S2. Univariate and multivariate analysis of factors associated with survival in CRC.

Supplementary Table S3. The proteins interacted with DDX39B were identified by Co-IP together with mass spectrometry (MS).

Supplementary Table S4. The energy component for the DDX39B-PKM2 complex.

Supplementary Table S5. Oligonucleotide sequences used in this study.

Supplementary Table S6. Reagent or resource used in this study.

Materials and Methods

**Cell culture**

Human CRC cell lines HT29, HCT116, SW480, SW620 and LoVo were purchased from American Type Culture Collection (ATCC), and were authenticated by short tandem repeat profiling. HT29 and LoVo cells were cultured using RPMI-1640 medium, and SW480, SW620, HCT116 were cultured using Dulbecco’s modified Eagle’s medium, and all cells were supplemented with 10% fetal bovine serum, 100 U/mL penicillin and 100 μg/mL streptomycin in a saturated humidity atmosphere of 5% CO_2_ at 37°C. Cell subculturing and cryopreservation were performed according to ATCC protocols. All cells were tested for mycoplasma contamination every six months and confirmed to be negative.

**Plasmid construction**

To generate the mammalian expression plasmids, full-length human DDX39B cDNA (NM_004640.7) was cloned into the pCMV-Tag 2B vector (Flag-tag) and pRK7 vector (Myc-tag); full-length human PKM2 cDNA (NM_002654.6) and PKM1 cDNA (NM_182470.4) were cloned into the pRK7 vector (Myc-tag) and pcDNA3.1 (HA-tag), respectively. The pBiFc-VN173 and pBiFc-VC155 plasmids were a gift from Chang-Deng Hu (Addgene plasmid #22010 and #22011). DDX39B and PKM2 cDNA were cloned into the bimolecular fluorescence complementation vectors, respectively. The plasmid, pCMV-Flag-ubiquitin, was kindly provided by Professor Hongbo Hu. To generate bacterial expression plasmids, full-length human DDX39B, STUB1 (NM_005861.4) and STAT3 (NM_139276.3) were cloned into the pGEX-5X-3 vector (fused with GST-tag), while PKM2 was cloned into the pET28a vector (fused with His-tag). The construction of mutant plasmids in this study was carried out using the Fast Mutagenesis System kit according to the user’s manual (TransGen Biotech). These plasmids pENTER-Sp1, pENTER-ETS1, pENTER-c-JUN, pGEX-5X-3-GST-importin α5, pGEX-5X-3-GST-β-catenin, pGL3-DDX39B promoter (wild type: -1500 / +60) were produced by Vigenebio.

**Infections and transfections**

All lentivirus in this study were purchased from VectorBuilder, Yunzhou Biosciences Co., Ltd. China. They are described briefly as follows, pLV-Puro-U6 vector containing shRNA-negative control (shNC), shRNA#1-DDX39B (sh#1), shRNA#2-DDX39B (sh#2), shRNA-PKM2; pLV-Neo-CMV vector containing luciferase; pLV-Puro-CMV vector containing Flag-DDX39B^WT^ or Flag-DDX39B^R319A^; pLV-Neo-CMV vector containing shPKM2-resistant PKM2^WT^ or PKM2^R399/400A^; pLV-Puro-U6-Terminator-CMV vector containing shPKM2 and DDX39B^WT^ together. Cells were infected by lentivirus with 4 μg/ml polybrene for three days. Stable cell lines were established by puromycin or G-418 selection. For transient transfections, cells were transduced with indicated plasmids or siRNA using lipofectamine 3000 (Invitrogen) according to the manufacturer’s protocol. The shRNA sequences are shown in **Supplementary Table 5**.

**Quantitative real-time PCR (qPCR)**

Total RNA was isolated using TRIzol reagent (CoWin Biosciences) following the manufacturer’s protocol and 0.5 μg total RNA per reaction was used to synthesize first strand cDNA using Superscript II (Invitrogen). Quantitative real-time PCR was performed with SYBR green qPCR master mix (Bimake), using a CFX Connect real-time PCR system (Bio-Rad). The primers are listed in **Supplementary Table 5**, and the relative expressions of mRNA were calculated by 2^‑∆∆Ct^ method and normalized to the internal control β-actin, according to a previous paper ^1^.

**Western blotting**

Cells were lysed with RIPA buffer containing protease and phosphatase inhibitors (TargetMol, C0004) in an ice-bath for 30 min. Equal aliquots of total protein were separated by SDS-PAGE on an 8%-12% gel, transferred to a PVDF membrane, and blocked with TBST buffer containing 5% fat-free milk for 1 h, after which the membranes were incubated with primary antibodies at the recommended dilution overnight at 4°C. Subsequently, the membranes were incubated with appropriate secondary antibodies conjugated with horseradish peroxidase (HRP) for 1 h, and protein bands were visualized with a chemiluminescent HRP substrate and imaging system (Chemidoc, Bio-Rad).

**Assessment of cell viability and proliferation**

For the CCK-8 assay, cells (5×10^3^ cells per well) were seeded into 96-well plates, and cultured for the indicated length of time. The assay was performed according to the manufacturer’s protocol (TargetMol, C0005) and the OD_450_ was measured. For clone formation assay, cells (600 cells per well) were seeded into 24-well plates and cultured for two weeks. Then cells were fixed in 4% formaldehyde, stained with 0.5% crystal violet, and cell clones were photographed and counted by ImageJ software. For the EdU assay, cells (1.5×10^4^ cells per well) were seeded into 96-well plates, and cultured for 24 h. Cells were stained with the Cell-Light EdU Apollo488 kit (RiboBio), nuclei with DAPI, and images were obtained with an inverted fluorescence microscope (Axio Observer D1/cam HRC, Zeiss). The proportion of EdU-positive cells was calculated using ImageJ software.

**Migration and invasion assays**

The migration and invasion assays were carried out with the use of 8.0 μM pore-size transwell chambers (Corning). For the migration assay, 5×10^4^ cells were resuspended in 200 μL serum-free DMEM medium and added into the upper chambers, while 1×10^5^ cells in chambers coated with growth-factor reduced Matrigel were used for the invasion assay. DMEM medium supplemented with 10% FBS was added to the bottom chamber. The migrated and invasive cells on the membranes were stained with 0.5% crystal violet, photographed under a microscope, and the numbers of cells were counted using ImageJ software.

**Wound-healing assay**

For the *in vitro* wound healing assay, the indicated cells were grown to confluency as monolayers, and then scratched with a pipette tip. The cells were washed with PBS to remove floating cells, and the scratch was imaged. Cells were then incubated in serum-free medium for 24 h or 48 h, imaged again and the percentage of the scratched area covered by cells was determined.

**Immunoprecipitation analysis**

Cells were lysed with lysis buffer (50 mM Tris-HCl, 150 mM NaCl, 1 mM EDTA, 1% Triton X-100, pH7.4) supplemented with protease inhibitor cocktail. Lysates were incubated with the indicated primary antibody and protein A/G magnetic beads or anti-FLAG M2 magnetic beads according to user instructions. Beads were washed three times with TBS buffer (50 mM Tris-HCl, 150 mM NaCl, pH 7.4), then boiled, and samples analyzed by western blotting.

**Chromatin immunoprecipitation**

Chromatin immunoprecipitation assays were performed on CRC cells using the EZ-Magna ChIP™ kit (Millipore) as described previously ^2^. Briefly, cells (1×10^7^ cells per condition) were crosslinked and lysed according to the manufacturer’s instructions. DNA fragments were immunoprecipitated by indicated antibody, and tested by qPCR analysis using the indicated primers (**Supplementary Table 5**).

**Dual luciferase reporter assay**

The transcription activity of human DDX39B promoter were examined using Dual-Luciferase Reporter Assay System (Promega) as described previously ^2^. Cells were transfected with DDX39B promoter reporter construct (0.25 μg) and pRL-TK (0.05 μg) with or without Sp1 plasmid (0.5 μg) using 2 μL lipofectamine 3000 per well in 48-well plates. Twenty-four hours post-transfection, cells were lysed and luciferase activity was detected according to the manufacturer’s instructions using a Multi-Mode Microplate Reader.

**Cycloheximide (CHX) chase assay**

The target cells (1×10^6^ cells per well) were seeded into 24-well plates, cultured for 24 h, and incubated with 75 μg/ml CHX for different times as indicated in figure 4e and supplemental figure 6i-j. Cells were then harvested and lysed for western blotting analysis.

**Immunofluorescence staining**

Cells grown on coverslips were fixed with 4% formaldehyde, permeabilized in 0.3% Triton X-100, blocked with 5% horse serum, and incubated with the indicated primary antibodies diluted as recommended in the user’s manual overnight at 4°C. After washing, the coverslips were incubated with the corresponding Alexa Fluor 488 and/or Alexa Fluor 594 secondary antibodies at a dilution of 1:1000 (Invitrogen). Nuclei were stained with DAPI and cells were imaged with a fluorescence microscope (Imager Z2, Zeiss).

**PKM2 oligomerization assay**

Lysates were prepared from cells disrupted by ultrasound, and the protein concentration of the lysates was approximately 4 mg/ml. Proteins were chemically cross-linked with 0.025% glutaraldehyde (GA) at 37°C for 3 min. The protein cross-linking reaction was immediately terminated by addition of 50 mM Tris-HCl, pH 8.0, and the oligomerization state of PKM2 was measured by western blotting.

**Molecular docking**

The crystal structures of DDX39B (PDB ID: 1XTI) and PKM2 (PDB ID: 1T5A) available from the Research Collaboratory for Structural Bioinformatics Protein Data Bank (RCSB PDB) were utilized for determining protein-protein docking through an online server (http://zdock.umassmed.edu/), and all docking parameters were set at default values. The top conformation was selected for molecular dynamic simulation. The binding free energy and energy component for the DDX39B-PKM2 complex were calculated using the trajectories of molecular dynamic simulation during 40 to 50 seconds.

**H&E staining and immunohistochemistry**

H&E staining and immunohistochemistry (IHC) analysis were carried out as previously described ^3^. For IHC, tissue sections were evaluated by intensity and extent of cell staining. The staining intensity was graded as: negative (0), slight (1), moderate (2), or strong (3), and the percentage of positive staining cells was calculated. The score (0-3) of each section was counted using the following weighting method: 0 × % of negative staining + 1 × % of slight staining + 2 × % of moderate staining + 3 × % of strong staining.

**References**

1. Li, K. *et al.* DDX17 nucleocytoplasmic shuttling promotes acquired gefitinib resistance in non-small cell lung cancer cells via activation of beta-catenin. *Cancer Lett.* **400**, 194-202 (2017).
2. Li, K. *et al.* ZNF32 protects against oxidative stress-induced apoptosis by modulating C1QBP transcription. *Oncotarget* **6**, 38107-38126 (2015).
3. Li, K. *et al.* Cytoplasmic expression, antibody production, and characterization of the novel zinc finger protein 637. *Appl. Microbiol. Biotechnol.* **97**, 741-749 (2013).

Figure. S1.


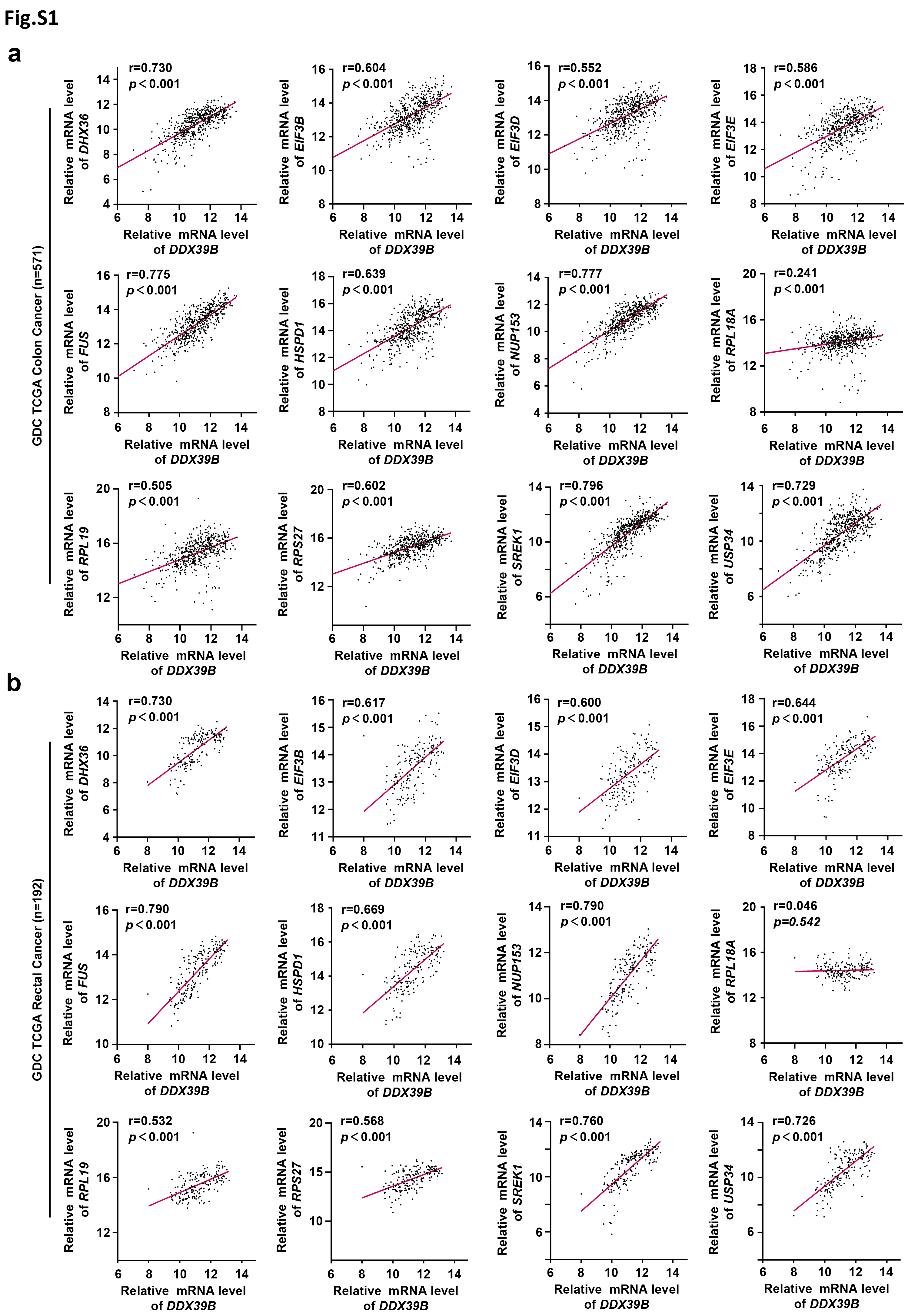


**Supplementary figure 1. The correlation of DDX39B with other subgroup genes in CRC patients. (a-b)** The correlation between DDX39B transcripts with DHX36, EIF3B, EIF3D, EIF3E, FUS, HSPD1, NUP153, RPL18A, RPL19, RPS27, SREK1 and USP34 were analyzed through an online tool (https://xenabrowser.net/). Data were obtained from GDC TCGA Colon Cancer (**a**) and GDC TCGA Rectal Cancer (**b**).

Figure. S2.


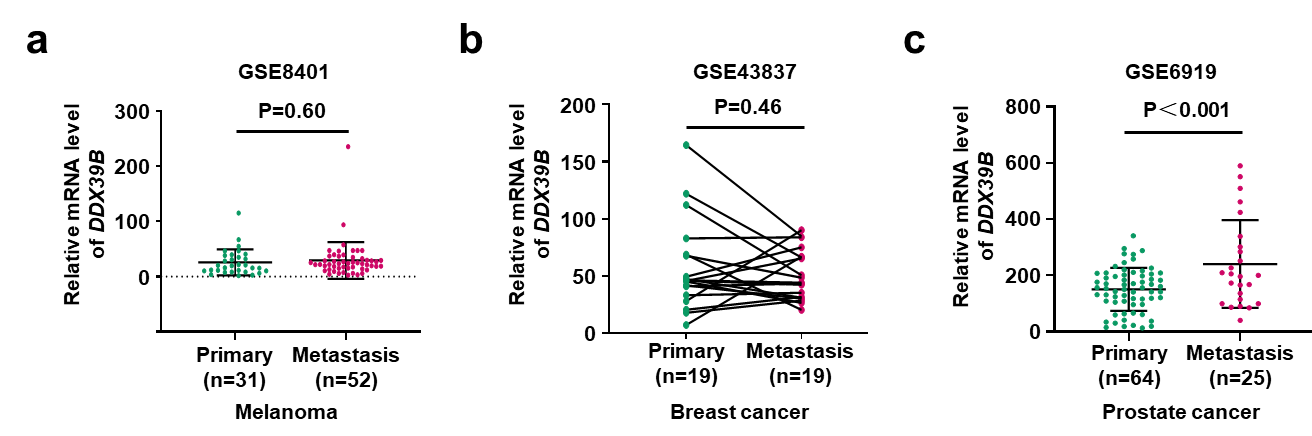


**Supplementary figure 2. The expression of DDX39B in** **primary and metastatic tumor tissues. (a-c)** DDX39B mRNA levels in primary and metastatic tumor tissues from melanoma (**a**), breast cancer (**b**) and prostate cancer (**c**) datasets were analyzed by two-tailed unpaired *t* test (**a, c**) or two-tailed paired *t* test (**b**).

Figure. S3.

**
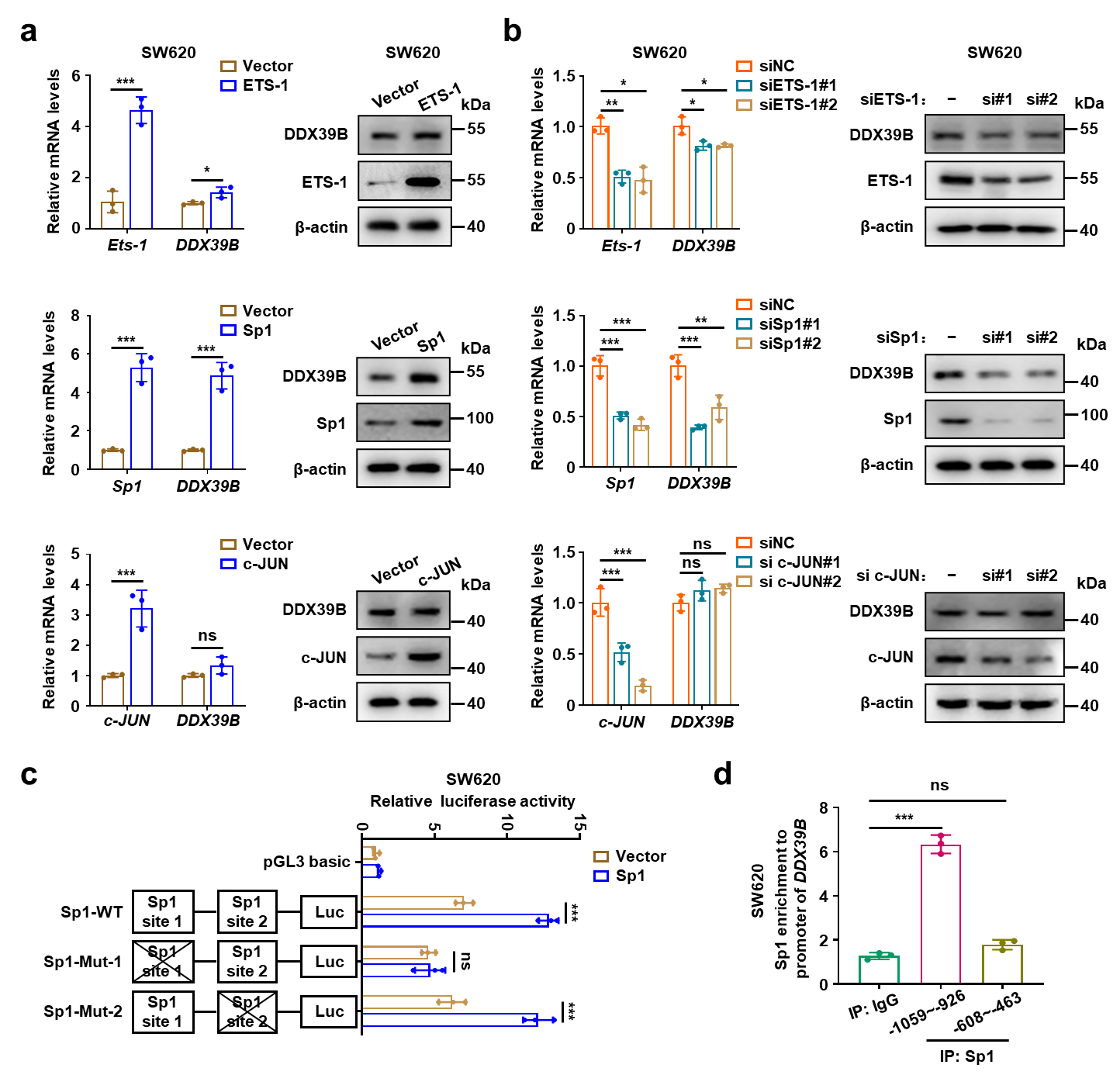
Supplementary figure 3.** **Sp1 activates DDX39B transcription in CRC cells.** (**a**) SW620 cells were transiently transfected with the plasmids of ETS-1, Sp1 or c-JUN, respectively. The mRNA and protein expressions of DDX39B were measured by qPCR and western blotting. (**b**) SW620 cells were transiently transfected with siRNAs targeting for ETS-1, Sp1 or c-JUN, respectively. DDX39B mRNA and protein levels were tested by qRT-PCR and western blotting. (**c**) The relative luciferase activity was detected in SW620 cells transfected with the indicated DDX39B promoter in the presence or absence of Sp1. (**d**) DNA fragments from SW620 cells were immunoprecipitated with the Sp1 specific antibody and analyzed by qRT-PCR using the indicated primers. Data are presented as mean ± SD. The *p* values were obtained by two-tailed unpaired t test (**a**) or one-way ANOVA (**b-d**). **p* < 0.05, ***p* < 0.01, ****p* < 0.001, ns: not significant.

Figure. S4.


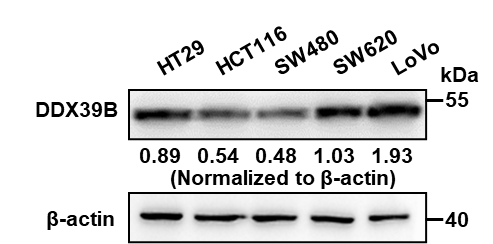


**Supplementary figure 4.** **DDX39B protein expression in CRC cell lines.** The DDX39B protein levels in CRC cell lines (HT29, HCT116, SW480, SW620 and LoVo) were detected by western blotting.

Figure. S5.


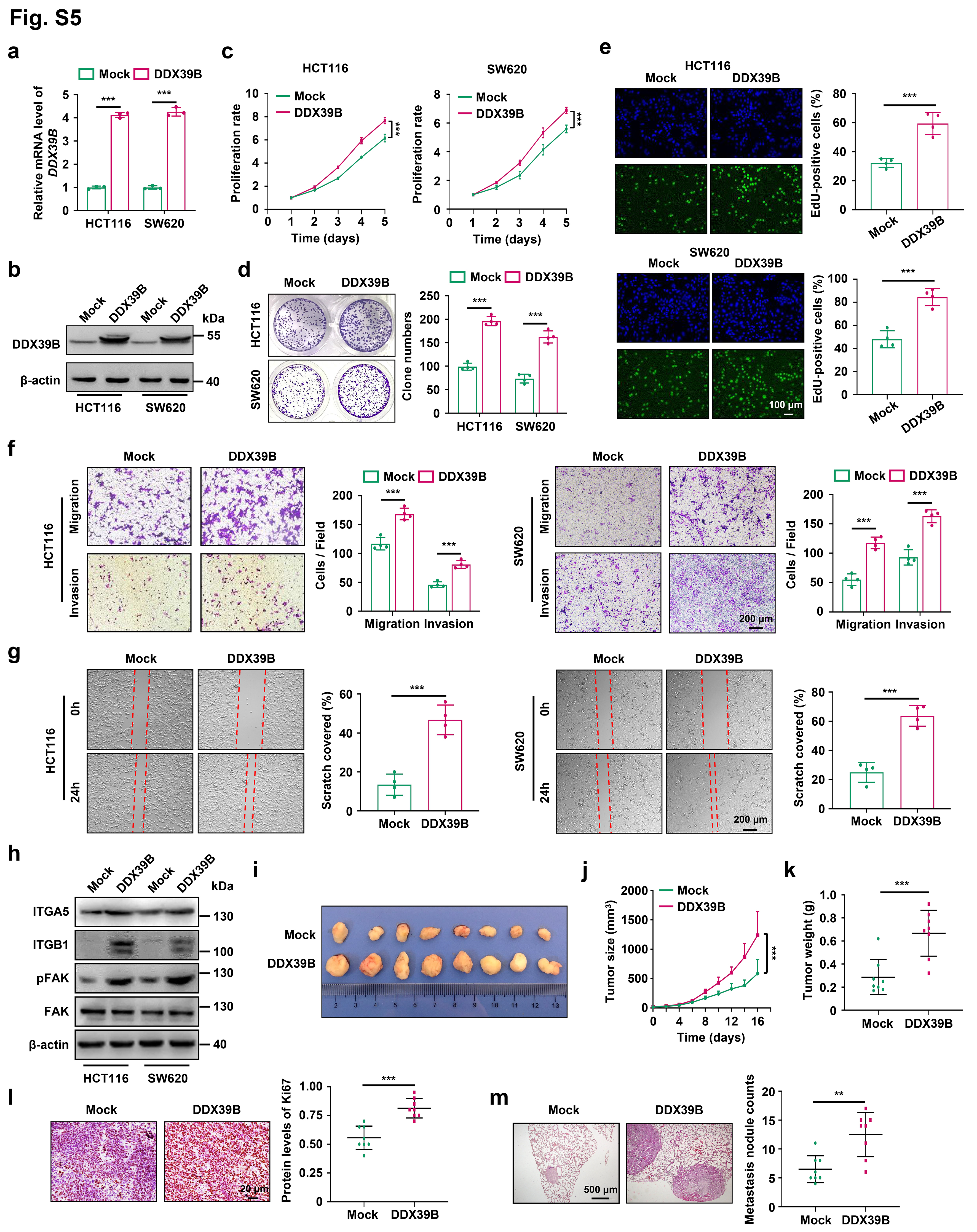


**Supplementary figure 5.** **DDX39B overexpression promotes CRC growth and metastasis. (a-b)** Overexpression efficiency mediated by lentivirus-delivered DDX39B cDNA was verified by qRT-PCR (**a**) and western blotting (**b**) in the indicated cell lines. (**c-e)** The viability and proliferation of CRC cells overexpressing DDX39B was measured by CCK8 assay (**c**), colony formation (**d**) and EdU assay (**e**). (**f-g)** The motility of DDX39B-overexpressing CRC cells was examined by transwell migration/invasion assays (**f**) and wound healing assays (**g**). (**h**) The indicated protein levels in DDX39B-overexpressing CRC cells were detected by western blotting. (**i-m)** The growth and metastatic abilities of HCT116 cells overexpressing DDX39B *in vivo* were assessed by subcutaneous xenograft and lung metastasis tumor models in nude mice (n = 8), respectively. The images (**i**), tumor sizes (**j**), tumor weights (**k**) and Ki-67 expressions (**l**) of the xenografts are shown. Representative pulmonary metastases detected by H&E staining are shown, along with the number of metastatic nodules (**m**). Data are presented as mean ± SD. The *p* values were obtained by two-way ANOVA (**c, j**) or Student’s *t* test (others). ***p* < 0.01, ****p* < 0.001.

Figure. S6.


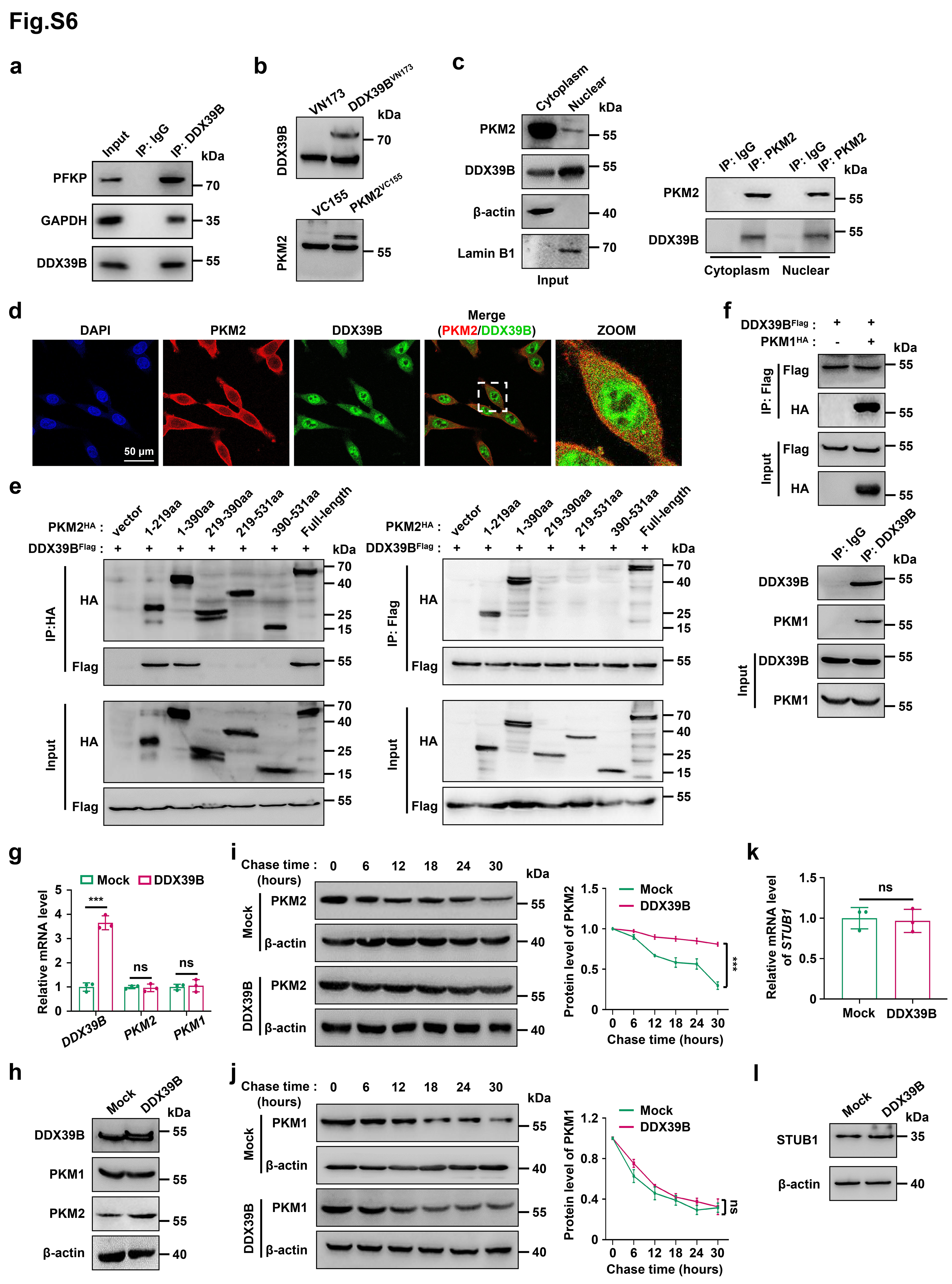


**Supplementary figure 6.** **DDX39B interacts with and stabilizes PKM2.** (**a**) The interaction between endogenous DDX39B with PFKP and GAPDH in HCT116 cells was detected by immunoprecipitation assay. (**b**) The bimolecular fluorescent plasmids (pBiFC-VN173-DDX39B and pBiFC-VC155-PKM2) were validated by western blotting. (**c**) Nuclear and cytosolic fractions from HCT116 cells were subjected to immunoprecipitation analysis. (**d**) The subcellular localization of endogenous DDX39B and PKM2 in HCT116 cells was visualized by immunofluorescence assay. (**e**) DDX39B^Flag^ and truncated PKM2^HA^ plasmids were co-transfected in HCT116 cells, and then subjected to immunoprecipitation assay. (**f**) The interaction between DDX39B and PKM1 in HCT116 cells was determined by immunoprecipitation analysis. (**g-h**) The mRNA and protein levels of PKM1 and PKM2 in DDX39B-overexpressing HCT116 cells were detected by qRT-PCR and western blotting, respectively. (**i-j**) The degradation rates of PKM2 and PKM1 protein in HCT116 cells overexpressing DDX39B were measured by cycloheximide (CHX) chase analysis. (**k-l**) The mRNA and protein levels of STUB1 in DDX39B-overexpressing HCT116 cells were detected by qRT-PCR and western blotting, respectively. Data are presented as mean ± SD. The *p* values were obtained by two-way ANOVA (**i, j**) or Student’s *t* test (**g, k**). ****p* < 0.001, ns: not significant.

Figure. S7.


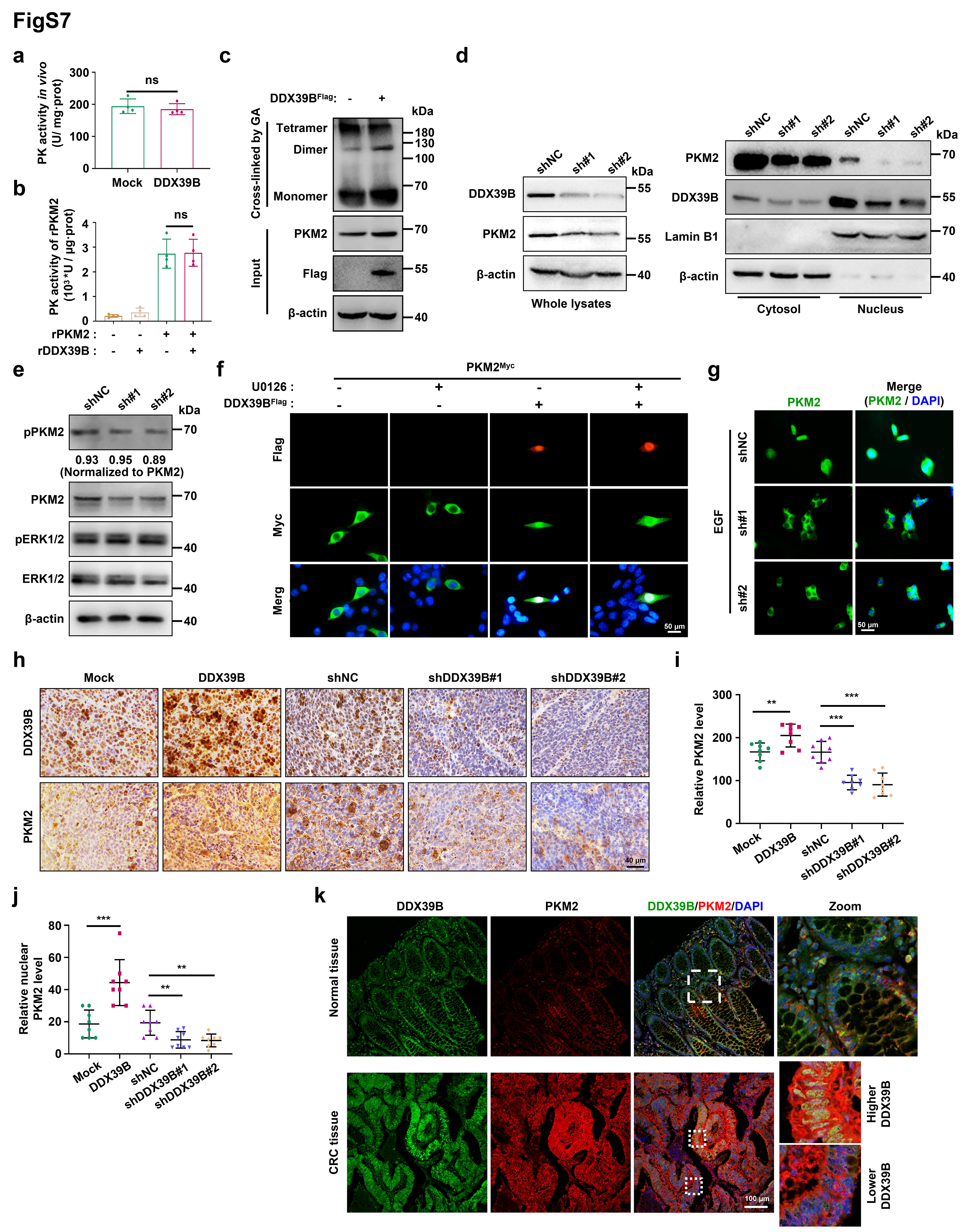


**Supplementary figure 7. DDX39B promotes ERK-independent PKM2 nuclear translocation.** (**a**) Lysates of HCT116 cells stably expressing mock or DDX39B were assayed for intracellular pyruvate kinase (PK) activity. (**b**) The *in vitro* PK activity of recombinant PKM2 (rPKM2) in the presence or absence of recombinant DDX39B (rDDX39B) was measured. (**c**) The oligomerization state of PKM2 upon DDX39B overexpression in HCT116 cells was determined by glutaraldehyde (GA) cross-linking assay. (**d**) The PKM2 protein levels in the whole-cell, nuclear and cytosolic lysates prepared from DDX39B-knockdown HCT116 cells were determined by western blotting. (**e**) The phosphorylation of PKM2^S37^ and ERK1^T202/Y204^, ERK2^T185/Y187^ in DDX39B-knockdown HCT116 cells was detected by western blotting. (**f**) HCT116 cells cotransfected with Myc-PKM2 and Flag-DDX39B were treated with 20 μM U0126 or vehicle, and the subcellular locations of DDX39B and PKM2 were visualized by immunofluorescence assay. (**g**) DDX39B-knockdown HCT116 cells were incubated with 150 ng/ml EGF, and the subcellular localization of PKM2 signals was observed by immunofluorescence assay. (**h-j**) The expression of DDX39B and PKM2 in xenograft tumors derived from the indicated DDX39B knockdown or overexpression- HCT116 cells were measured by immunohistochemistry assay. Representative images were shown (**h**), and the relative staining intensities of PKM2 or nuclear PKM2 in indicated group were measured (**i, j**). (**k**) The expression and subcellular localization of DDX39B and PKM2 in normal and CRC tissues was visualized by immunofluorescence assay. Data are presented as mean ± SD. The *p* values were obtained by Student’s *t* test (**a**) or one-way ANOVA (**b,** **i, j**). ***p* < 0.01, ****p* < 0.001, ns: not significant.

Figure. S8.


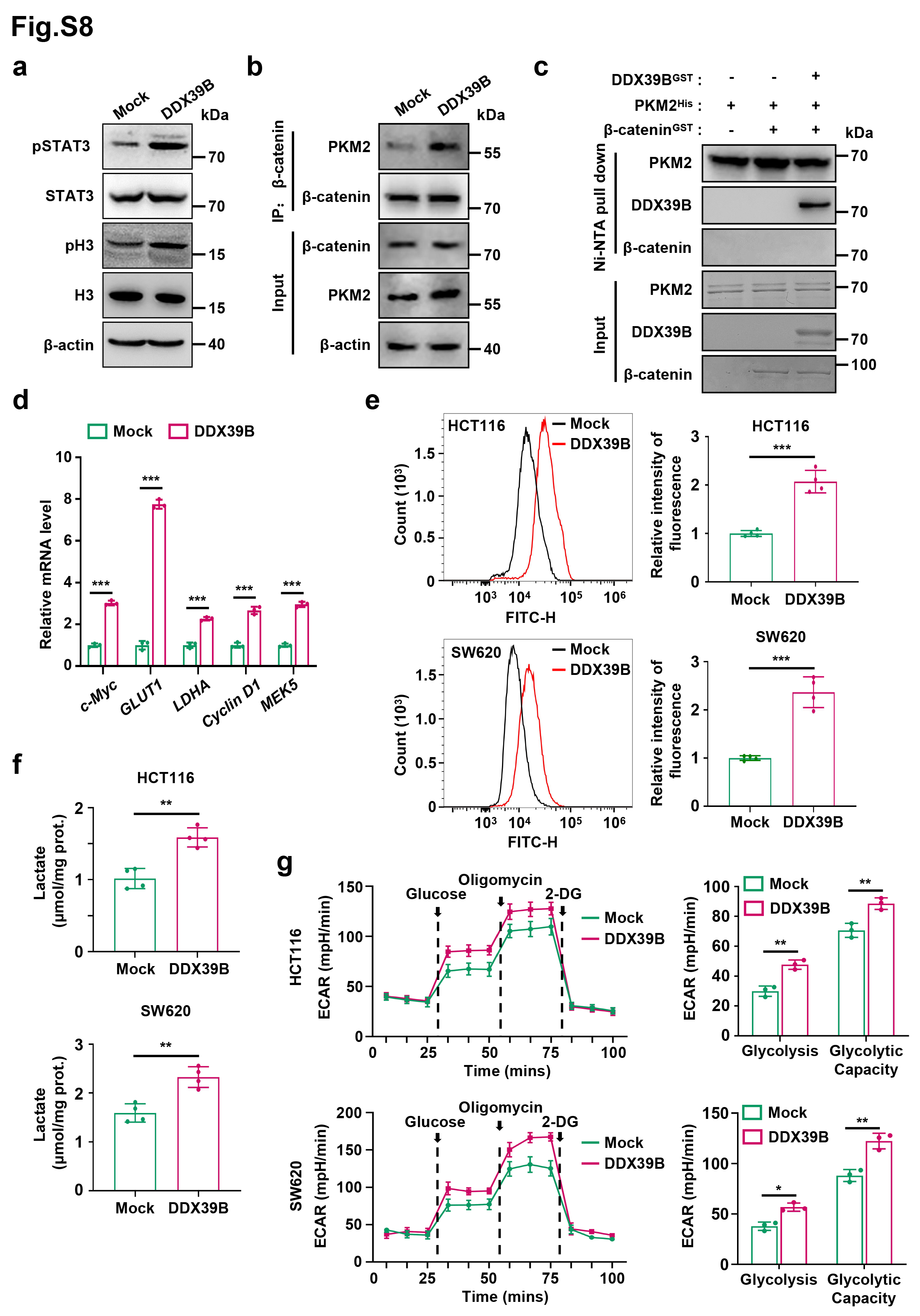


**Supplementary figure 8. DDX39B enhances nuclear PKM2 function and aerobic glycolysis in CRC cells.** (**a**) Phosphorylation of STAT3^Y705^ and histone H3^T11^ in DDX39B-overexpression HCT116 cells were detected by western blotting. (**b**) Association of PKM2 with β-catenin upon DDX39B overexpression was evaluated by immunoprecipitation in HCT116 cells. (**c**) *In vitro* binding analysis was performed using the indicated purified proteins. (**d**) The relative mRNA levels of c-Myc, GLUT1, LDHA, Cyclin D1 and MEK5 were measured by qPCR in HCT116 cells stably expressing mock or DDX39B. (**e**) The glucose uptake of DDX39B-overexpressing CRC cells was quantified by flow cytometry detection of the fluorescent glucose analogue, 2-NBDG. (**f**) The lactate level in CRC cells overexpressing DDX39B was quantified. (**g**) The extracellular acidification rate (ECAR) in DDX39B-overexpressing CRC cells was monitored, and the level of glycolysis and glycolytic capacity were calculated. Data are presented as mean ± SD. The *p* values were determined by Student’s *t* test. **p* < 0.05, ***p* < 0.01, ****p* < 0.001.

Figure. S9.


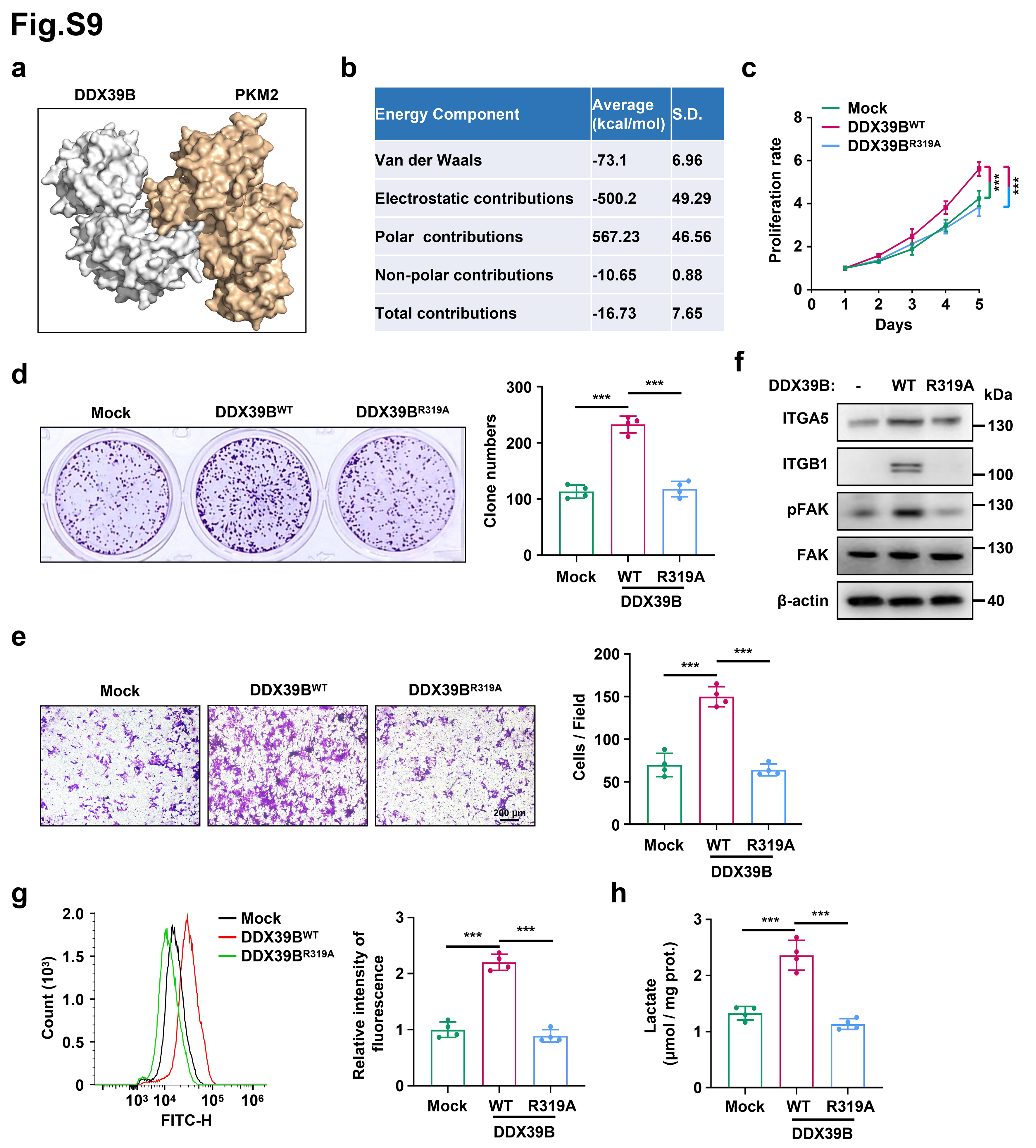


**Supplementary figure 9. Arg319 of DDX39B is required for PKM2 binding and retains the ability of DDX39B to promote carcinogenesis and metastasis in CRC.** (**a**) Primary conformation model for molecular docking of DDX39B with PKM2. (**b**) Binding free energy and energy component for DDX39B-PKM2 complex. (**c-h**) HCT116 cells were modified to stably express mock, DDX39B^WT^ or DDX39B^R319A^. (**c**) Cell viability was measured by CCK8 assay. (**d**) Cell growth was determined by colony formation assay. (**e**) Cell motility was determined by transwell migration assay. (**e**) The indicated protein levels were tested by western blotting. (**g**) Glucose uptake and (**h**) lactate production were measured. Data are presented as mean ± SD. The *p* values were determined using two-way ANOVA (**c**) or one-way ANOVA (others). ****p* < 0.001.

Figure. S10.


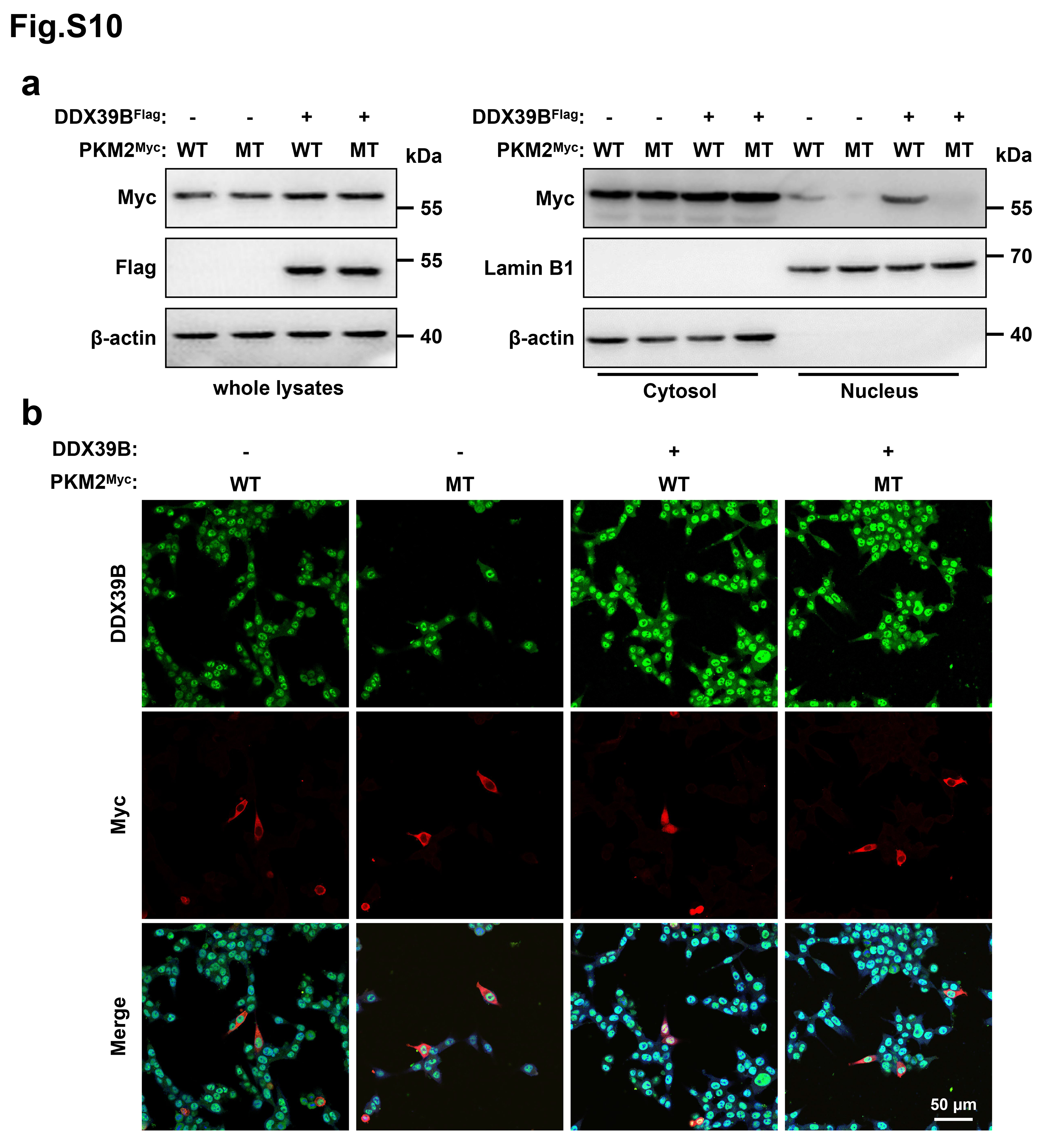


**Supplementary figure 10.** **DDX39B promotes the nuclear translocation of PKM2^WT^, but not PKM2^R399/400A^.** (**a-b**) HCT116 cells stably expressing mock or DDX39B were transfected with PKM2^WT^ or PKM2^R399/400A^ mutant (MT). (**a**) The Myc-PKM2 levels in the whole-cell, nuclear and cytosolic lysates were determined by western blotting. (**b**) Subcellular localization of Myc-PKM2 signals was observed by immunofluorescence assay.

Figure. S11.


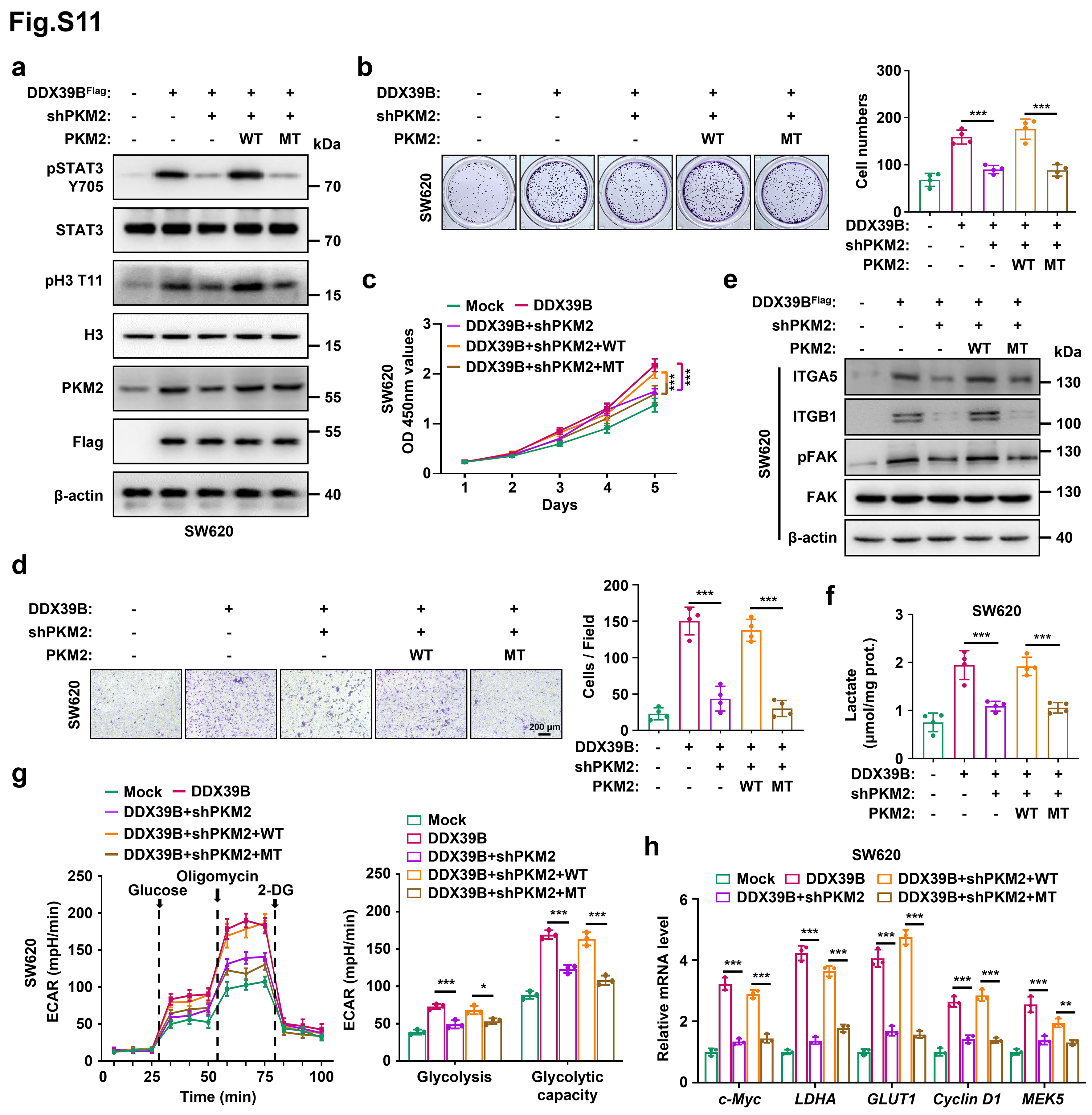


**Supplementary figure 11.** **Blocking PKM2 nuclear accumulation impairs DDX39B-triggered Warburg effect and tumorigenicity *in vitro*.** (**a-h**) SW620 cells simultaneously expressing DDX39B cDNA and PKM2-shRNA were re-introduced into RNAi-resistant PKM2^WT^ or PKM2^R399/400A^ mutant (MT), respectively. (**a**) Phosphorylation of STAT3^Y705^ and histone H3^T11^ was detected by western blotting. (**b**) The cell proliferation was measured by colony formation assay. (**c**) Cell viability was measured by CCK8 assay. (**d**) Cell motility was determined by transwell migration assays. (**e**) The indicated protein levels were detected by western blotting. (**f**) The lactate production in indicated SW620 cells was quantified. (**g**) The extracellular acidification rate (ECAR) of indicated SW620 cells was monitored, and the levels of glycolysis and glycolytic capacity were calculated. (**h**) The relative transcriptions of c-Myc, GLUT1, LDHA, Cyclin D1 and MEK5 were measured by qPCR. Data are presented as mean ± SD. The *p* values were obtained by two-way ANOVA (**c**) or one-way ANOVA (others). **p* < 0.05, ***p* < 0.01, ****p* < 0.001.

Figure. S12.


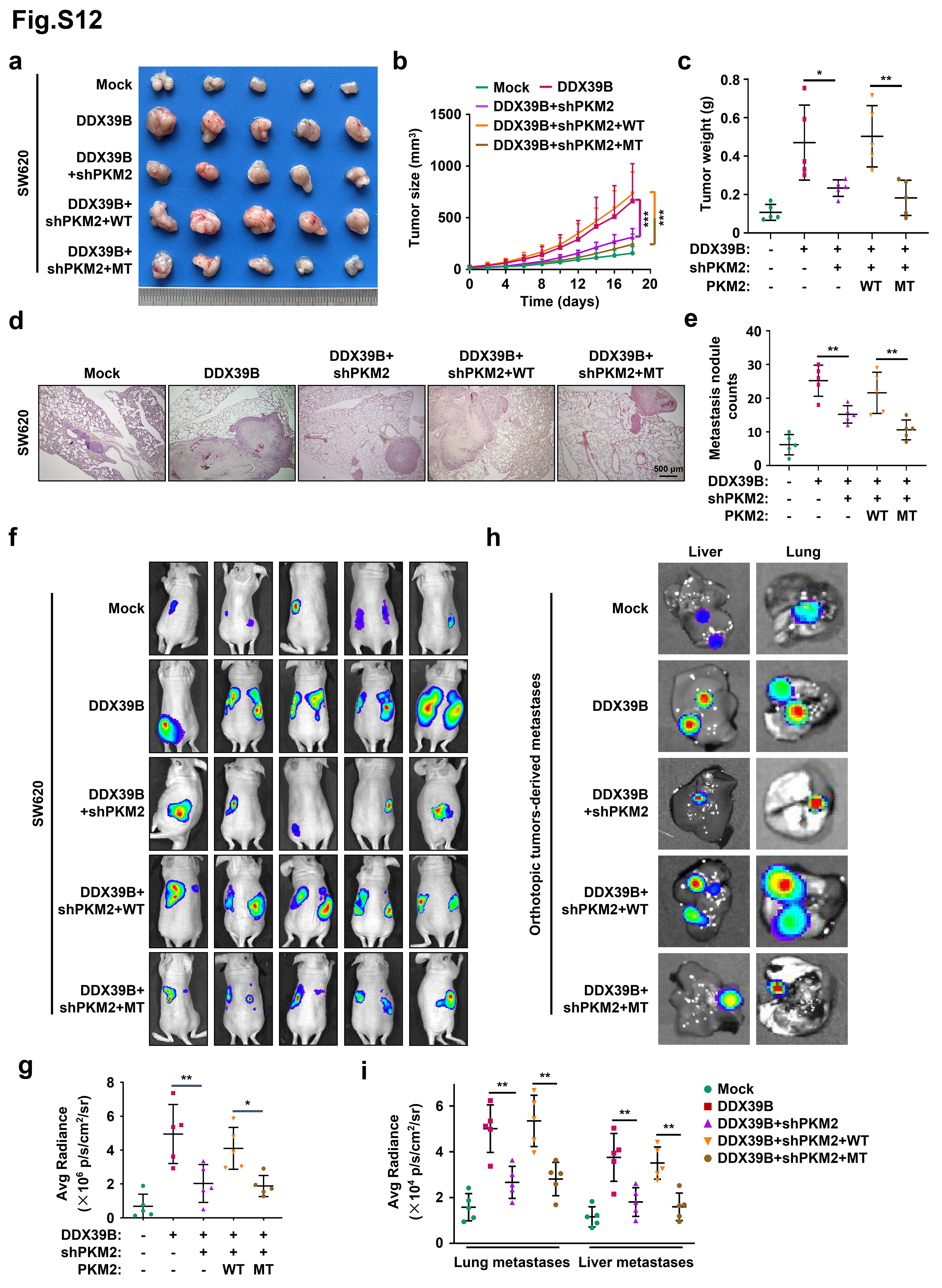


**Supplementary figure 12. Blocking PKM2 nuclear accumulation impairs DDX39B-triggered Warburg effect and tumorigenicity *in vivo*.** (**a-i**) SW620 cells simultaneously expressing DDX39B cDNA and PKM2-shRNA were re-introduced into RNAi-resistant PKM2^WT^ or PKM2^R399/400A^ mutant (MT), respectively. (**a-e**) The growth and metastatic abilities of indicated SW620 cells *in vivo* were assessed in nude mice by subcutaneous and lung metastasis tumor models (n = 5), respectively. The images (**a**), tumor sizes (**b**), and tumor weights (**c**) of subcutaneous xenografts are presented. Representative pulmonary metastases detected by H&E staining are shown (**d**), along with the number of metastatic nodules (**e**). (**f-i**) Indicated SW620 cells were orthotopically inoculated into the cecum of mice (n = 5). At day 60 after inoculation, the bioluminescent images of orthotopic tumors were captured (**f**) and light emissions were quantified (**g**). The representative bioluminescent images of the isolated lungs and livers were obtained (**h**), and the metastases were quantified (**i**). Data are presented as mean ± SD. The *p* values were obtained by two-way ANOVA (**b**) or one-way ANOVA (others). **p* < 0.05, ***p* < 0.01, ****p* < 0.001.

Figure. S13.


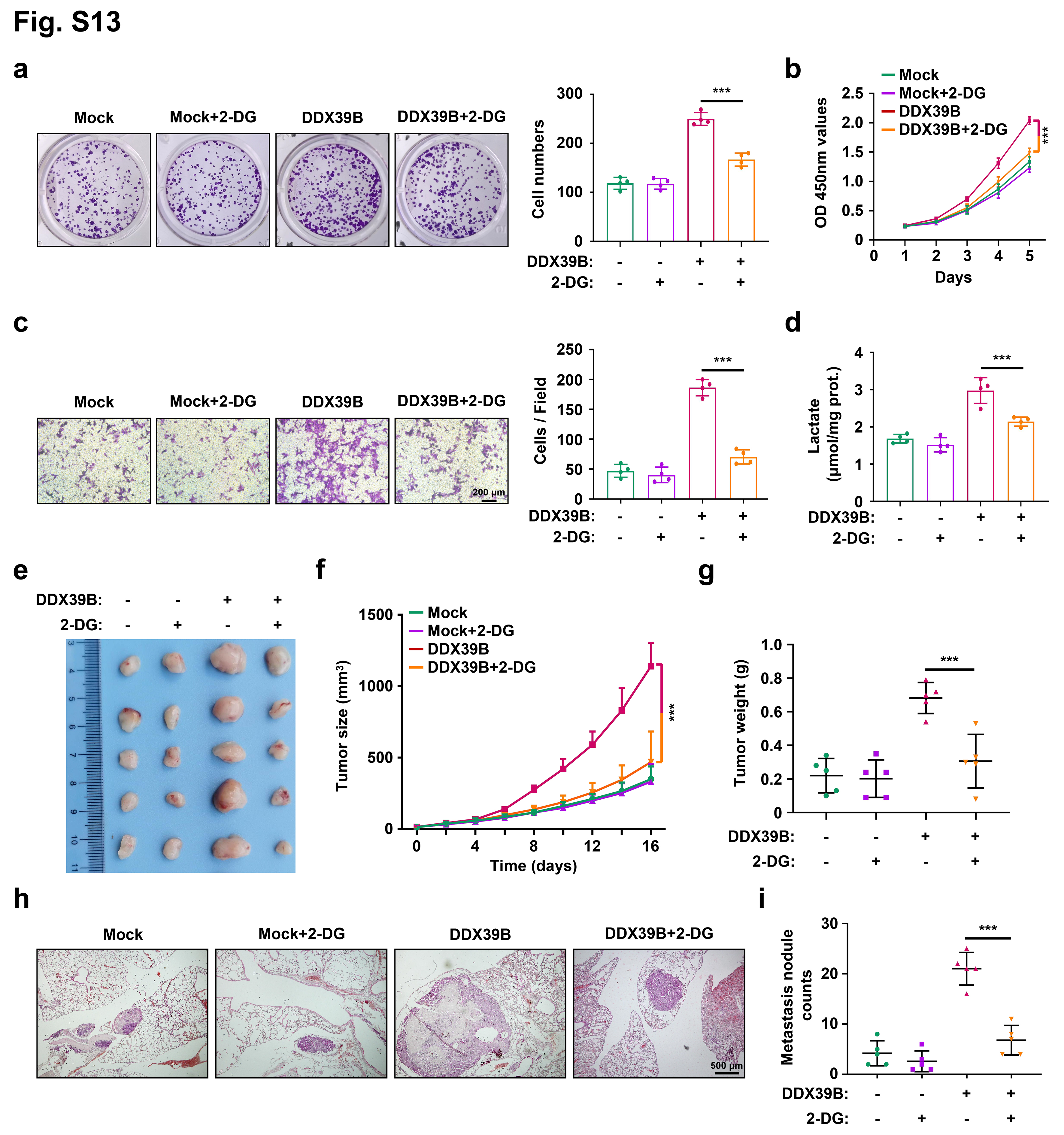


**Supplementary figure 13. Suppression of aerobic glycolysis impedes DDX39B-mediated tumorigenicity and progression in CRC.** (**a-d**) HCT116 cells stably expressing mock or DDX39B were incubated with or without the aerobic glycolysis inhibitor (2-DG, 5mM), and tested for colony formation (**a**), cell viability (**b**), migration (**c**), and lactate production (**d**). (**e-i**) The growth and metastatic abilities of indicated cells *in vivo* were assessed in nude mice by subcutaneous and lung metastasis tumor models (n = 5), respectively. One week after subcutaneous or tail-vein injection of HCT116 cells, the mice were intraperitoneally injected with 2-DG (100 mg/kg per mouse) or saline once a day for a week. The images (**e**), sizes (**f**) and weights (**g**) of subcutaneous xenografts are presented. Representative pulmonary metastases detected by H&E staining are shown (**h**), along with the number of metastatic nodules (**i**). Data are presented as mean ± SD. The *p* values were determined using two-way ANOVA (**b, f**) or one-way ANOVA (others). **p* < 0.05, ****p* < 0.001.
