# Supplementary figures and images for: DDX39B drives colorectal cancer progression by promoting the stability and nuclear translocation of PKM2
Source: Signal Transduct Target Ther. 2022 Aug 17;7:275. doi: 10.1038/s41392-022-01096-7 (PMC9381590; doi:10.1038/s41392-022-01096-7)

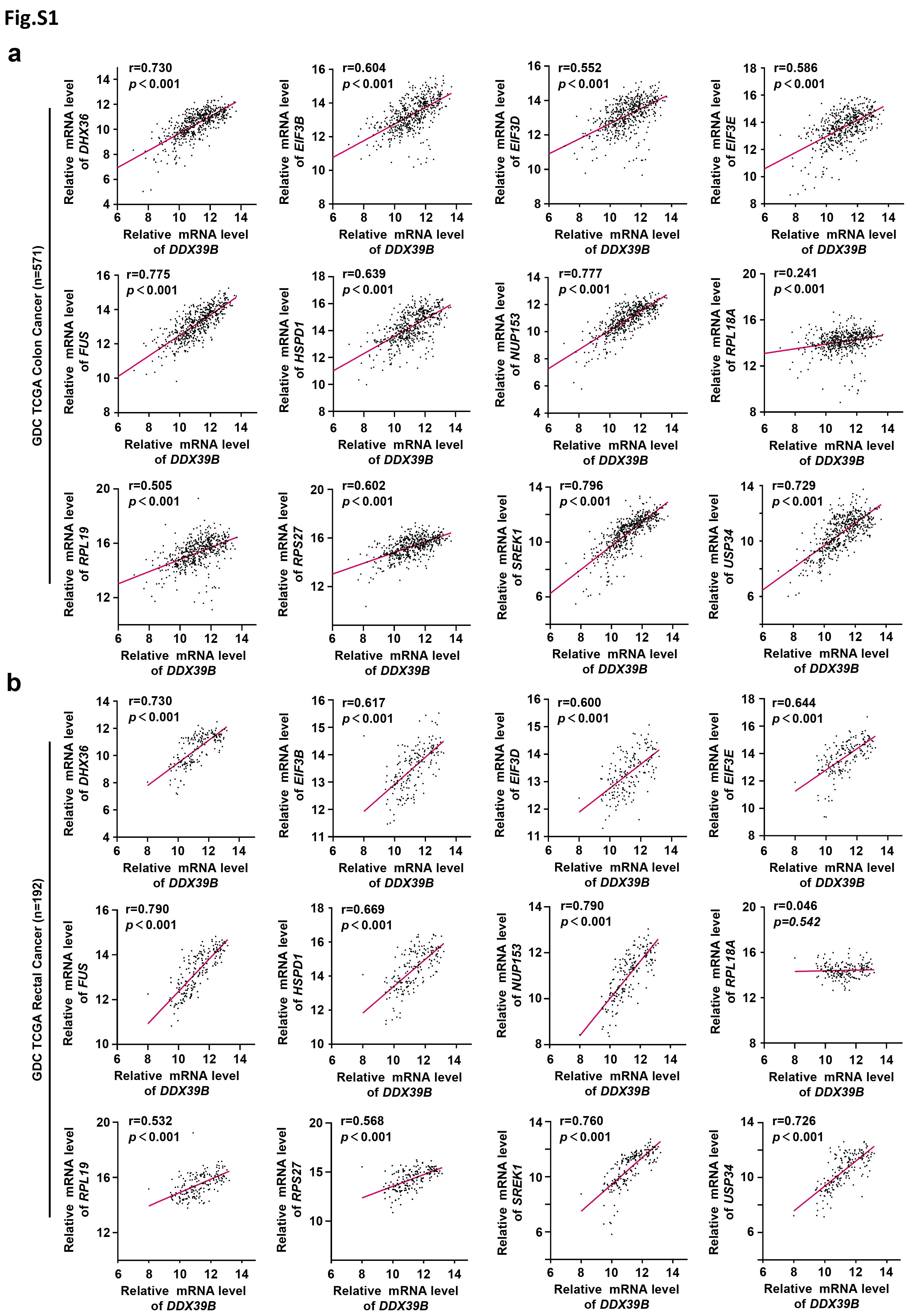

Supplement: Supplementary file 2 — Supplemental Figure 1 [file 41392_2022_1096_MOESM2_ESM.jpg]

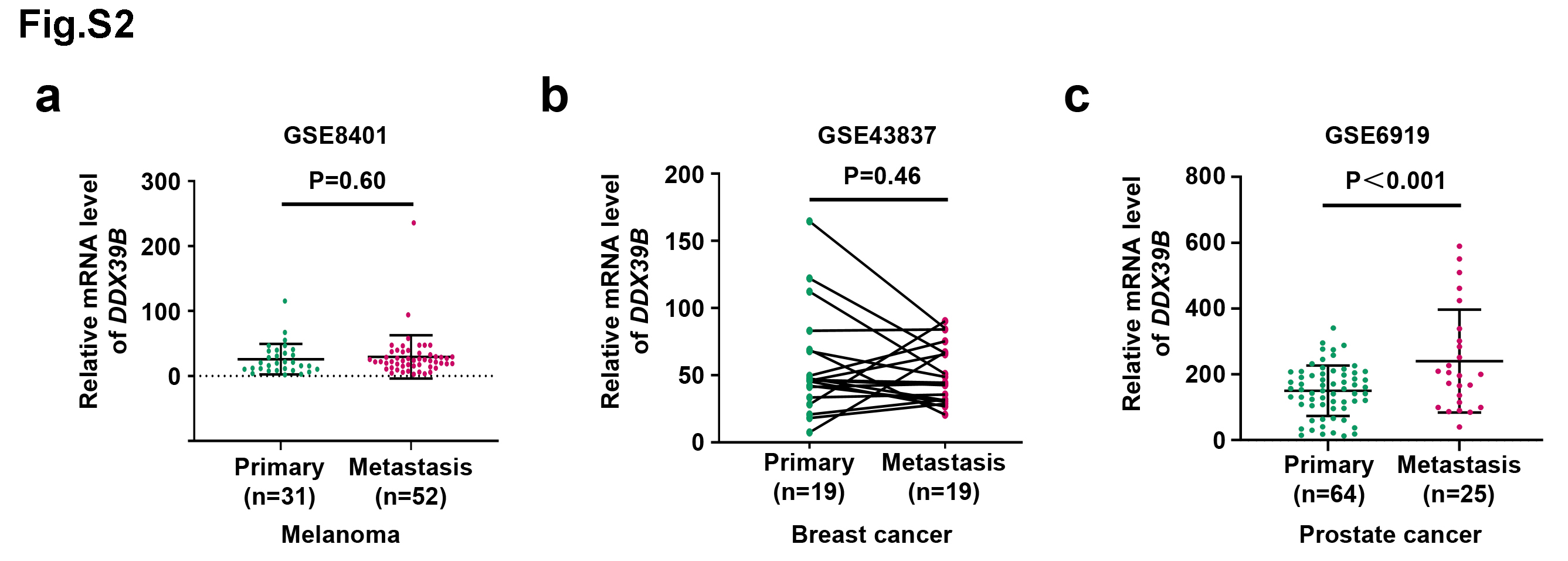

Supplement: Supplementary file 3 — Supplemental Figure 2 [file 41392_2022_1096_MOESM3_ESM.jpg]

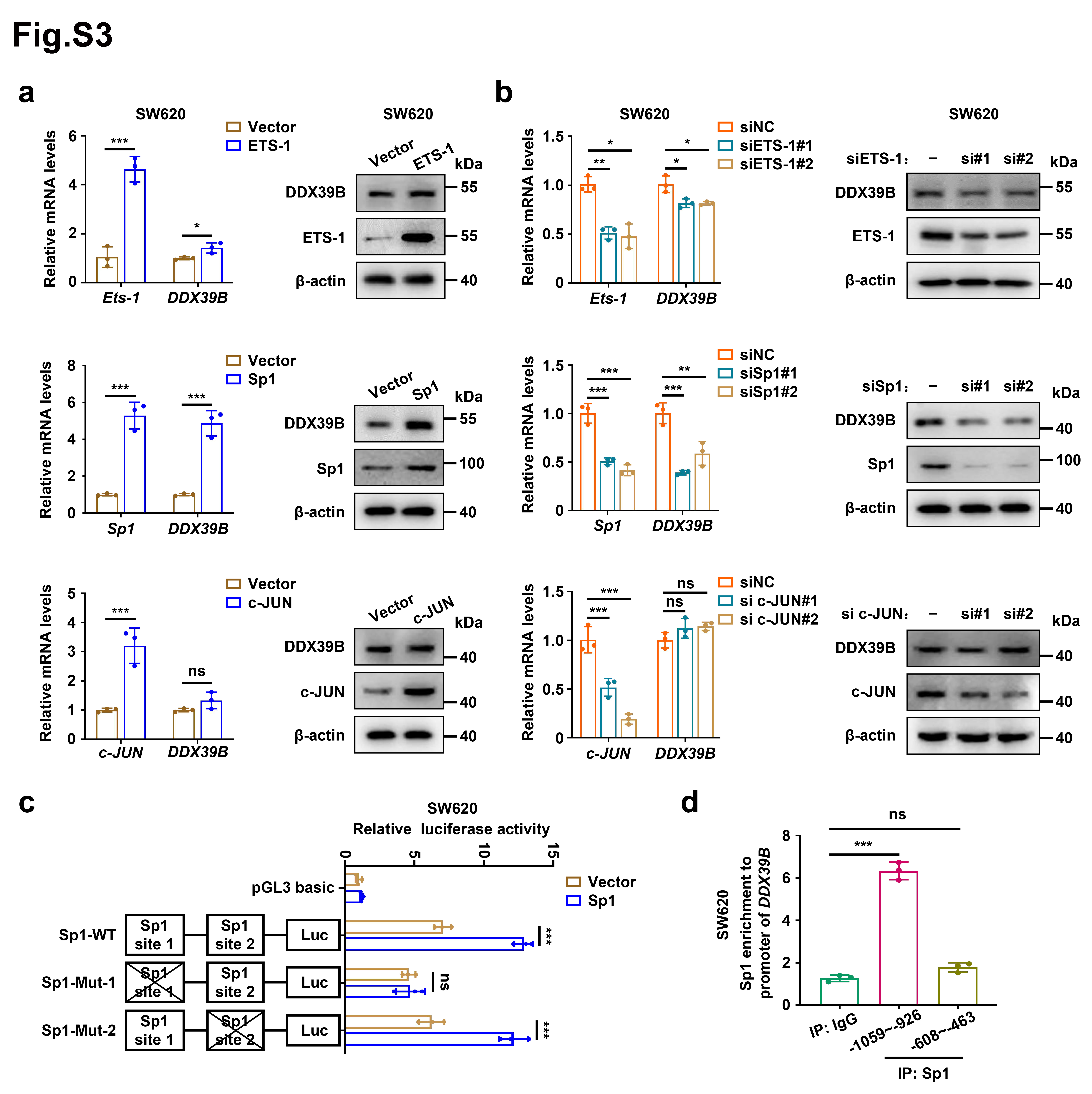

Supplement: Supplementary file 4 — Supplemental Figure 3 [file 41392_2022_1096_MOESM4_ESM.jpg]

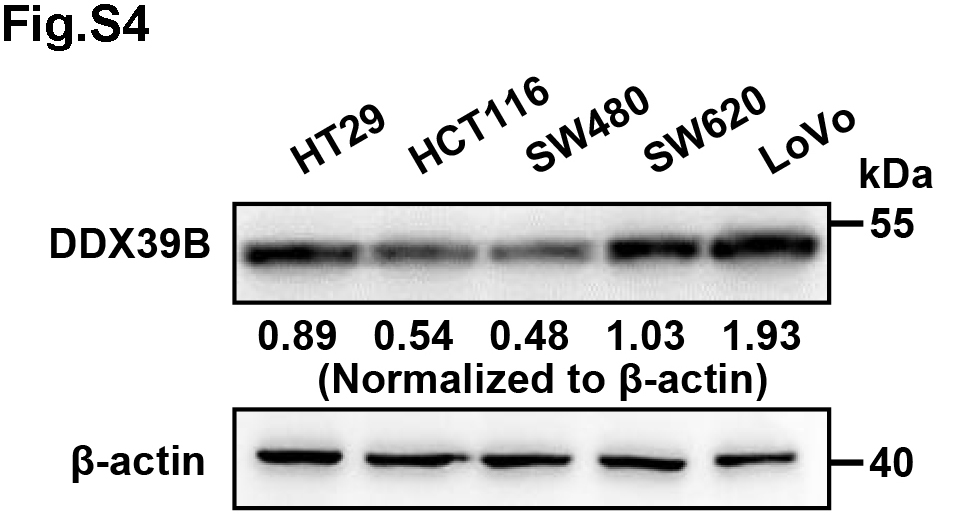

Supplement: Supplementary file 5 — Supplemental Figure 4 [file 41392_2022_1096_MOESM5_ESM.jpg]

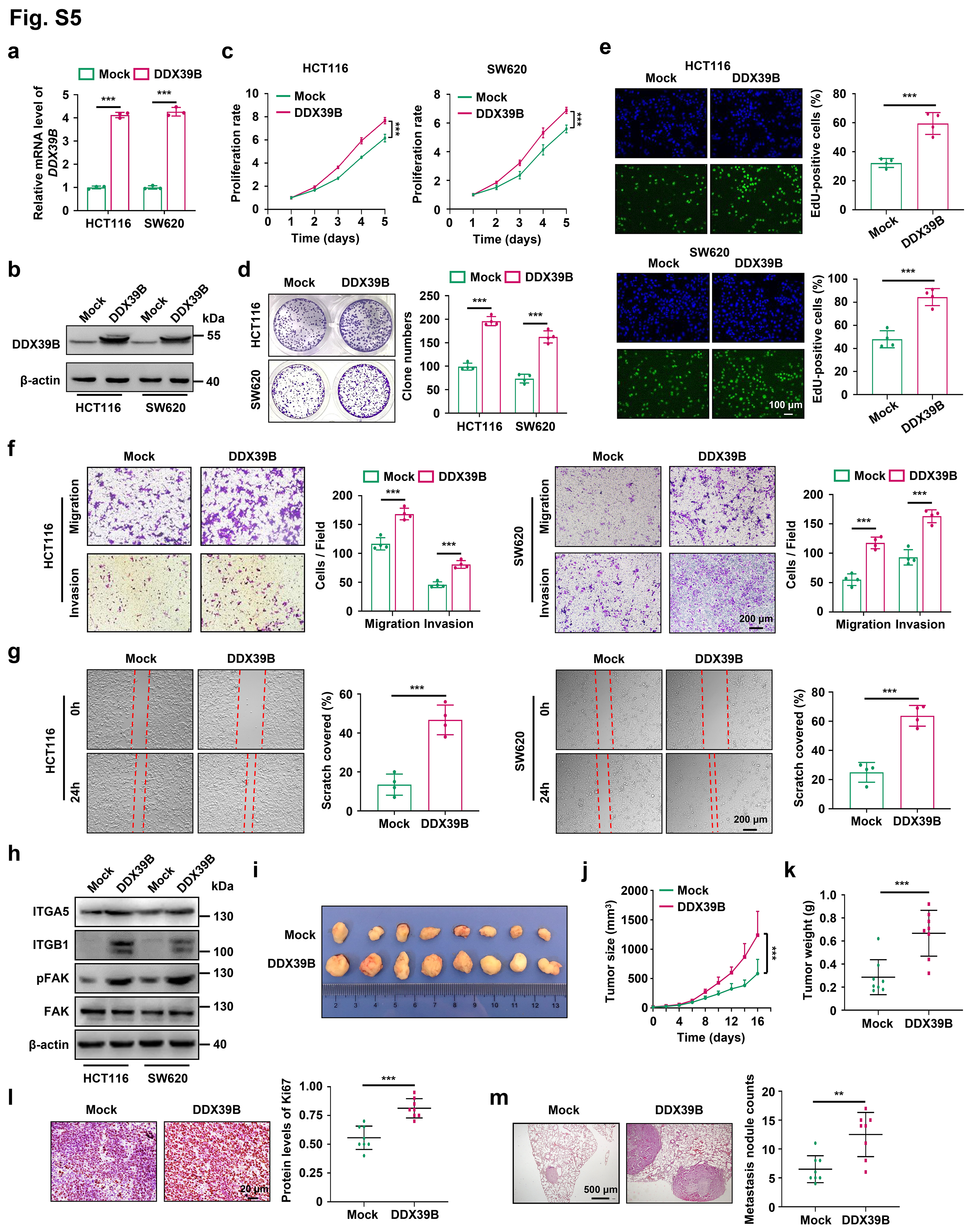

Supplement: Supplementary file 6 — Supplemental Figure 5 [file 41392_2022_1096_MOESM6_ESM.jpg]

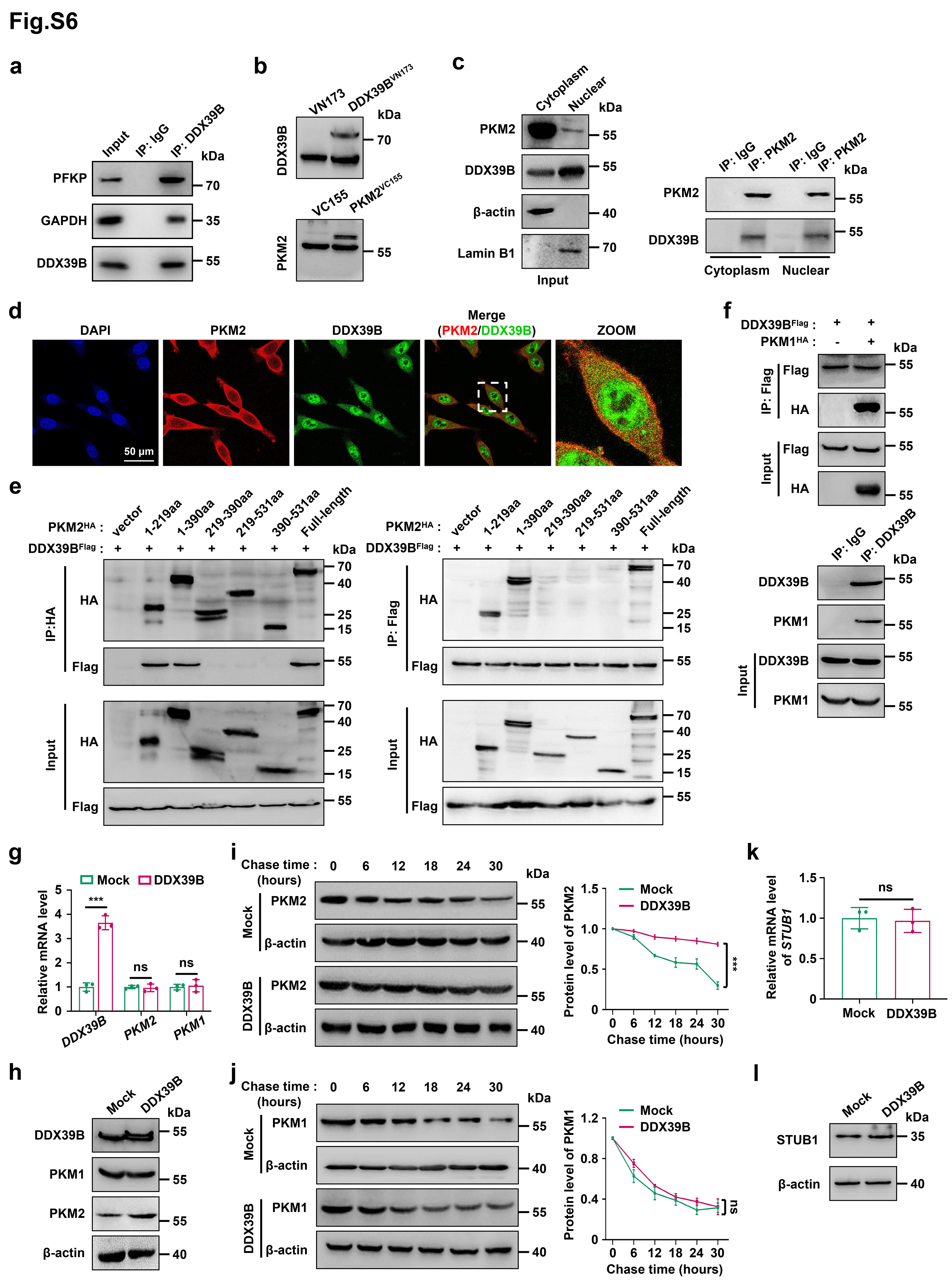

Supplement: Supplementary file 7 — Supplemental Figure 6 [file 41392_2022_1096_MOESM7_ESM.jpg]

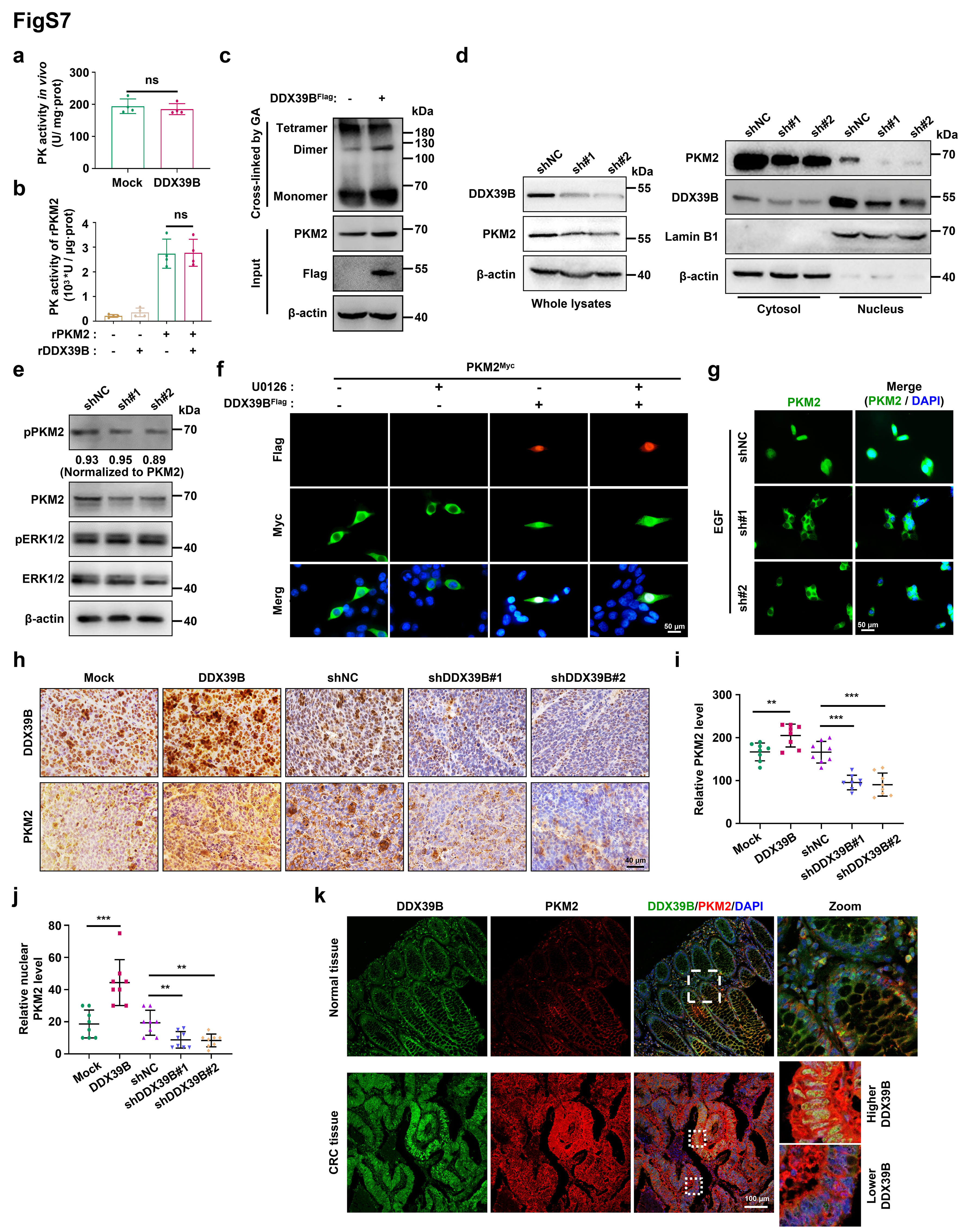

Supplement: Supplementary file 8 — Supplemental Figure 7 [file 41392_2022_1096_MOESM8_ESM.jpg]

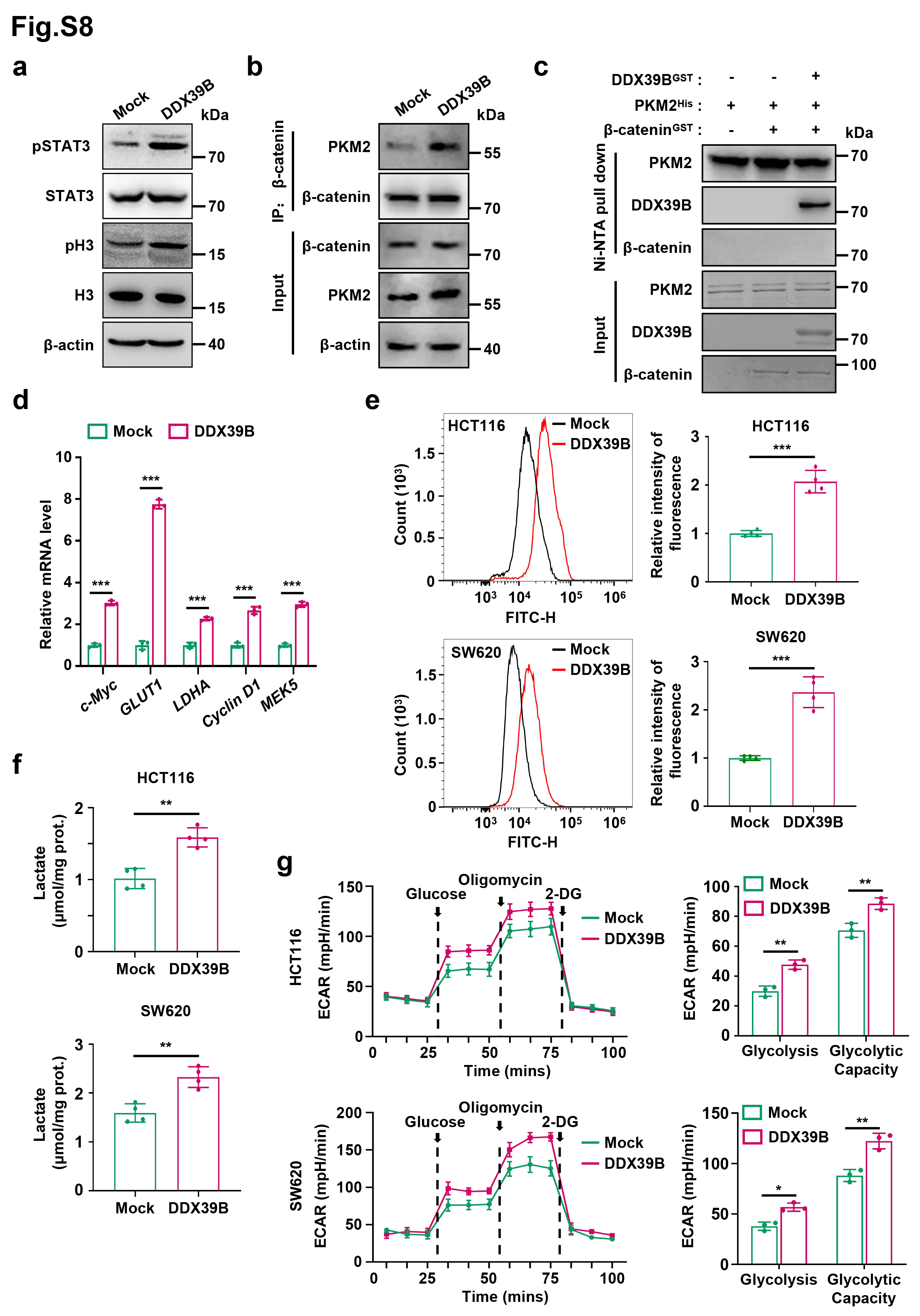

Supplement: Supplementary file 9 — Supplemental Figure 8 [file 41392_2022_1096_MOESM9_ESM.jpg]

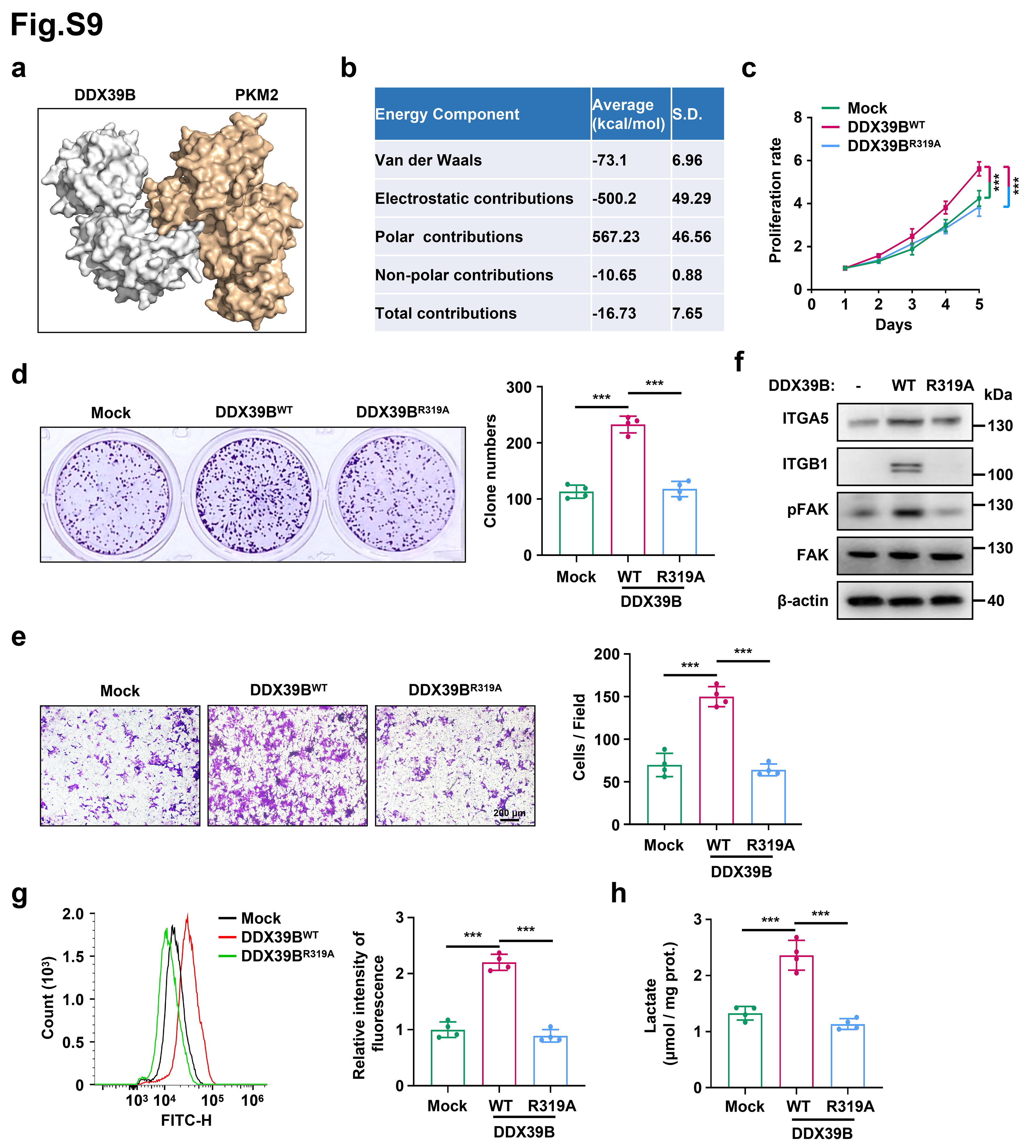

Supplement: Supplementary file 10 — Supplemental Figure 9 [file 41392_2022_1096_MOESM10_ESM.jpg]

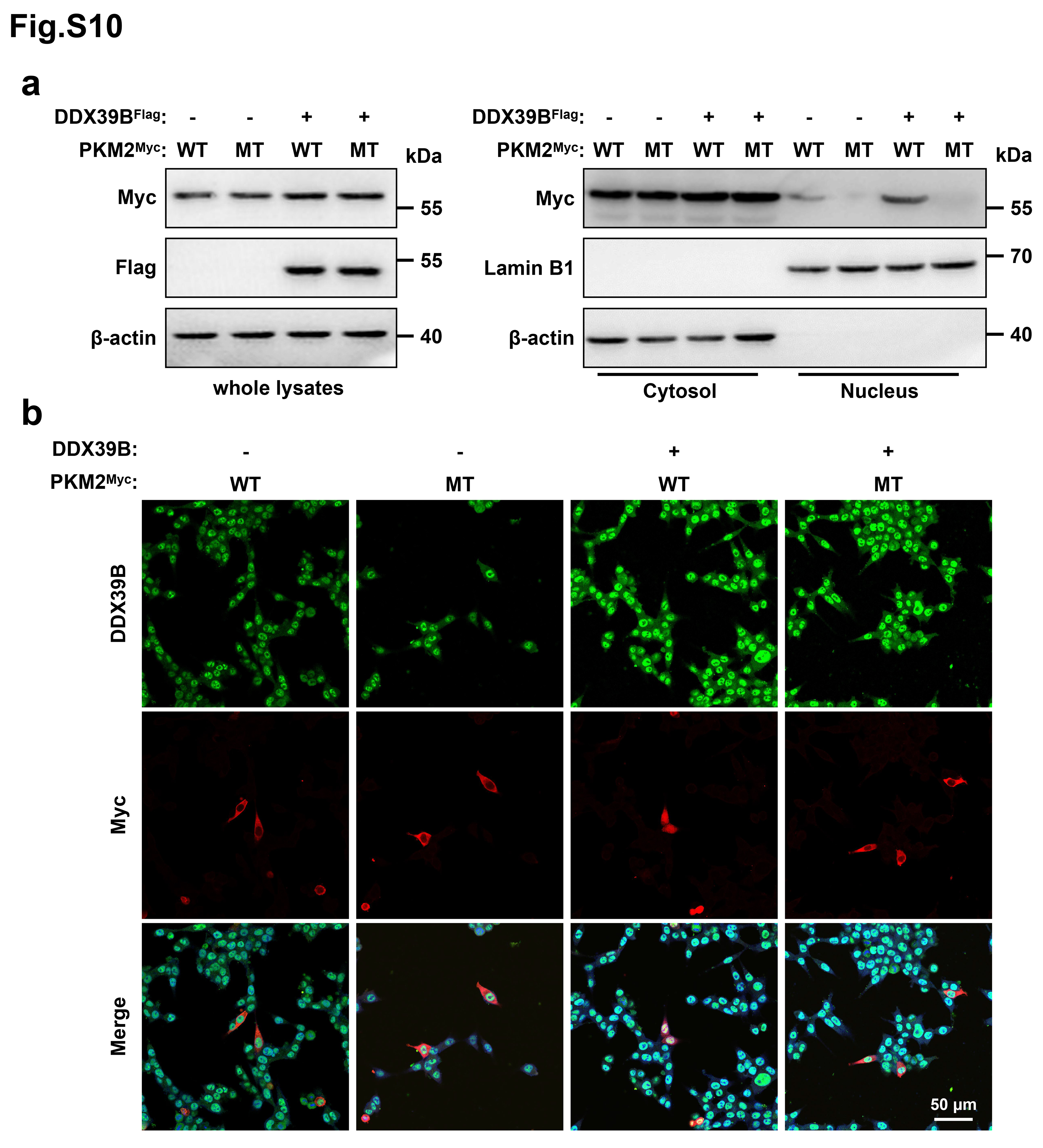

Supplement: Supplementary file 11 — Supplemental Figure 10 [file 41392_2022_1096_MOESM11_ESM.jpg]

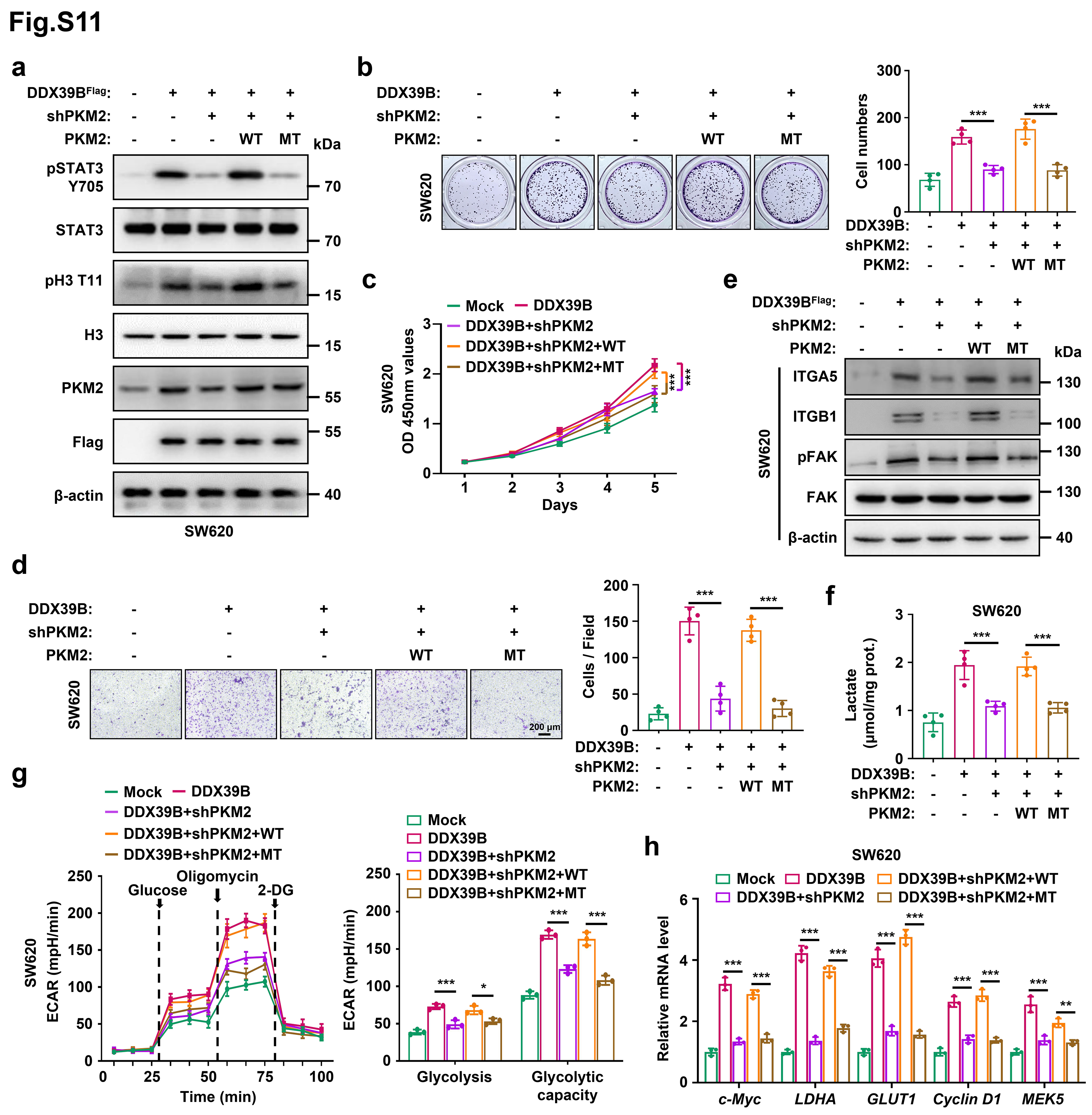

Supplement: Supplementary file 12 — Supplemental Figure 11 [file 41392_2022_1096_MOESM12_ESM.jpg]

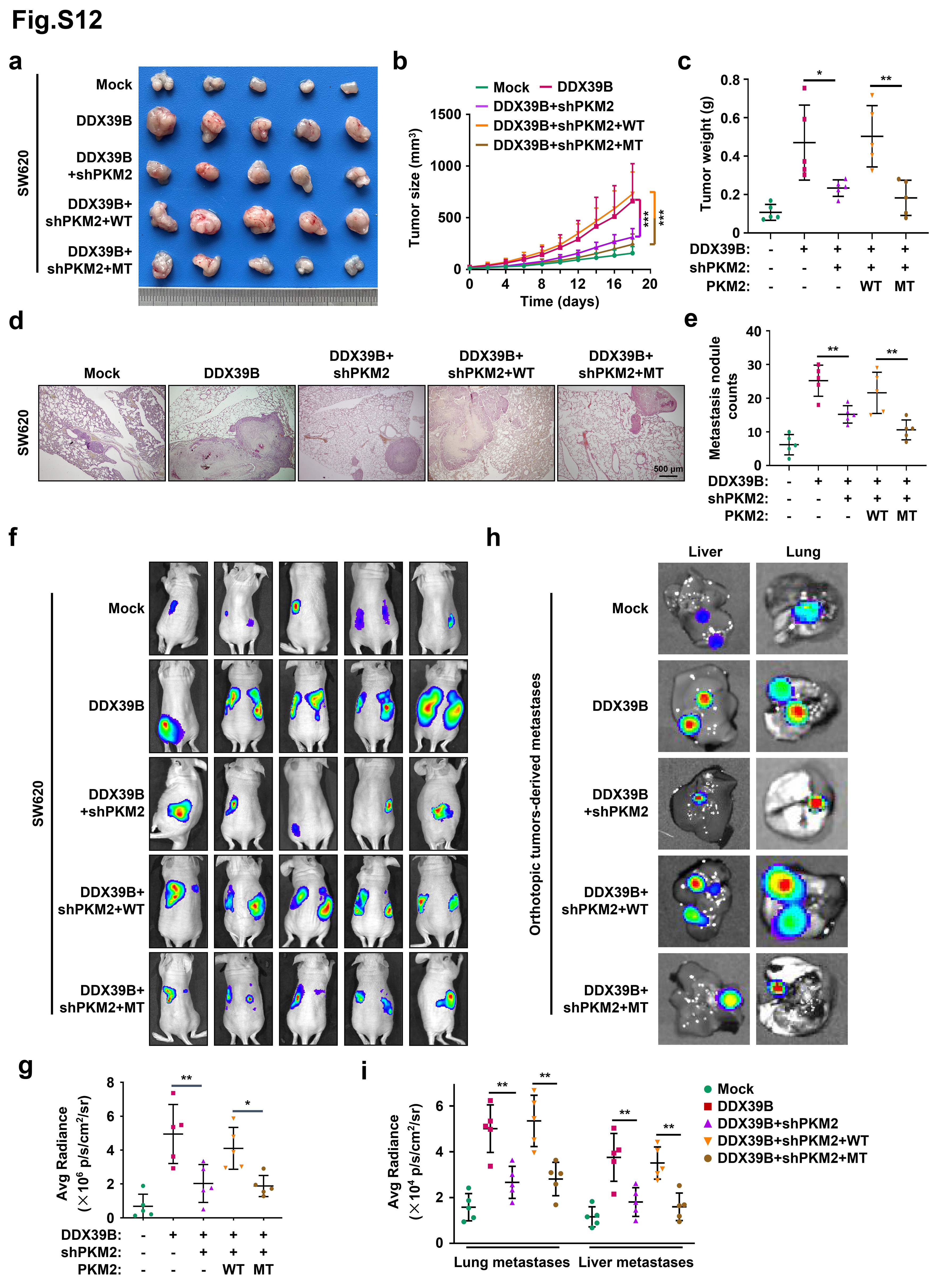

Supplement: Supplementary file 13 — Supplemental Figure 12 [file 41392_2022_1096_MOESM13_ESM.jpg]

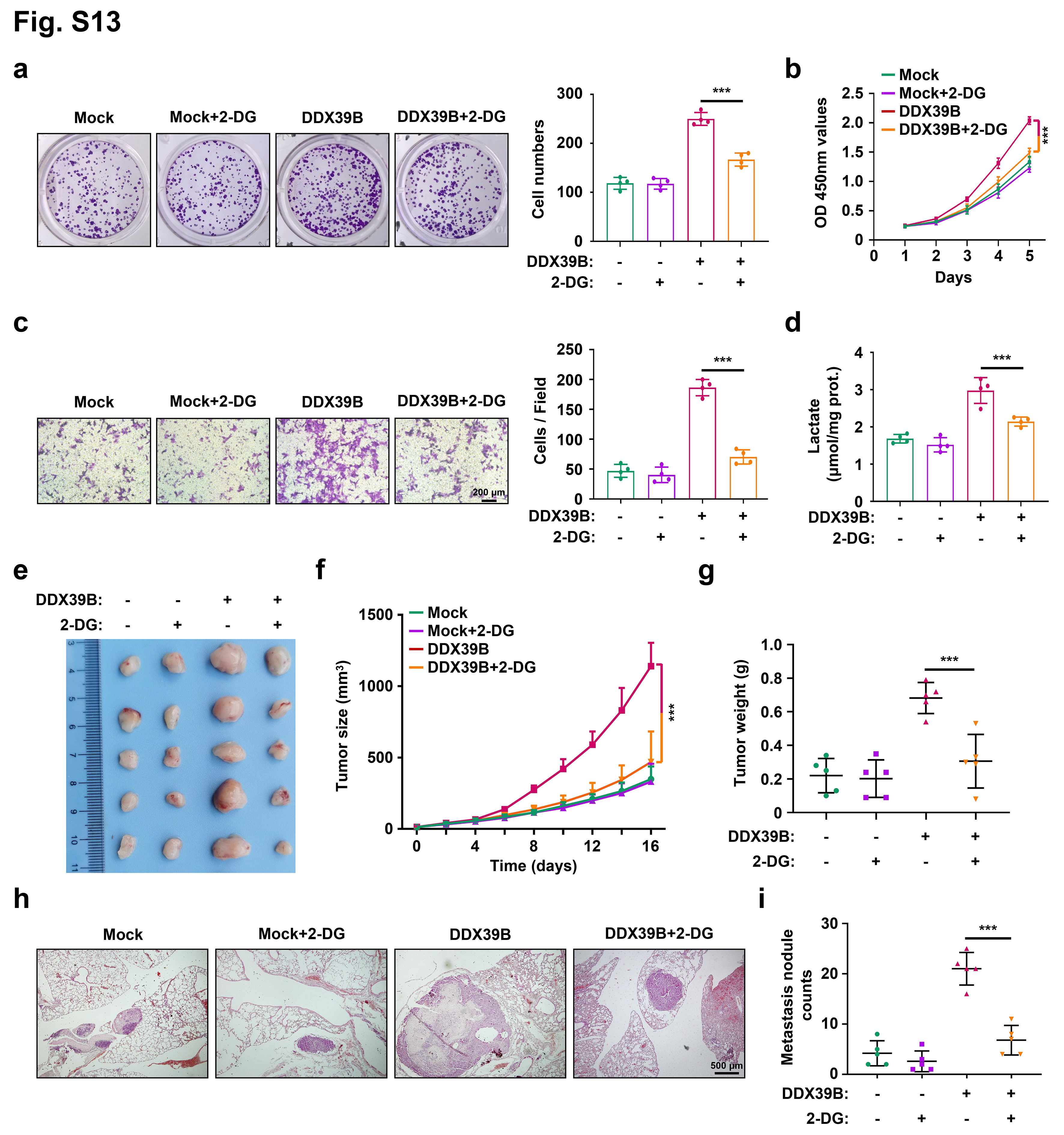

Supplement: Supplementary file 14 — Supplemental Figure 13 [file 41392_2022_1096_MOESM14_ESM.jpg]
