# Supplementary material for: DDX39B drives colorectal cancer progression by promoting the stability and nuclear translocation of PKM2
Source: Signal Transduct Target Ther. 2022 Aug 17;7:275. doi: 10.1038/s41392-022-01096-7 (PMC9381590; doi:10.1038/s41392-022-01096-7)
Supplement: Supplementary file 15 — Supplemental Table 1 [file 41392_2022_1096_MOESM15_ESM.docx]

**Supplemental Table 1.** Correlation between DDX39B expression and clinicopathological characteristics of CRC patients.

| Parameters | Cases  (n = 110) | DDX39B expression | | χ^2^ | *p* values |
| --- | --- | --- | --- | --- | --- |
|  |  | Low (n = 58) | High (n = 52) |  |  |
| Age (years) |  |  |  | 0.298 | 0.585 |
| <60 | 41 | 23 | 18 |  |  |
| ≥60 | 69 | 35 | 34 |  |  |
| Gender |  |  |  | 1.094 | 0.296 |
| Female | 43 | 20 | 23 |  |  |
| Male | 67 | 38 | 29 |  |  |
| Tumor location |  |  |  | 1.781 | 0.410 |
| Right colon | 25 | 15 | 10 |  |  |
| Left colon | 31 | 18 | 13 |  |  |
| Rectum | 54 | 25 | 29 |  |  |
| Tumor size |  |  |  | 15.476 | <0.001 |
| <5 cm | 40 | 31 | 9 |  |  |
| ≥5 cm | 70 | 27 | 43 |  |  |
| Histological grade |  |  |  | 15.326 | <0.001 |
| Well or moderate | 84 | 53 | 31 |  |  |
| Poor | 26 | 5 | 21 |  |  |
| Tumor invasion |  |  |  | 12.056 | 0.007 |
| T1 | 3 | 3 | 0 |  |  |
| T2 | 17 | 13 | 4 |  |  |
| T3 | 61 | 33 | 28 |  |  |
| T4 | 29 | 9 | 20 |  |  |
| Lymph node invasion |  |  |  | 17.448 | <0.001 |
| Absent | 66 | 46 | 20 |  |  |
| Present | 44 | 12 | 32 |  |  |
| Distant metastasis |  |  |  | 7.014 | 0.008 |
| Absent | 104 | 58 | 46 |  |  |
| Present | 6 | 0 | 6 |  |  |
| AJCC stage^a^ |  |  |  | 24.806 | <0.001 |
| Stage I | 19 | 16 | 3 |  |  |
| Stage II | 48 | 31 | 17 |  |  |
| Stage III | 37 | 11 | 26 |  |  |
| Stage IV | 6 | 0 | 6 |  |  |

AJCC, American Joint Committee on Cancer.

^a^AJCC Cancer Staging Manual, Eighth Edition.
