# Supplementary material for: DDX39B drives colorectal cancer progression by promoting the stability and nuclear translocation of PKM2
Source: Signal Transduct Target Ther. 2022 Aug 17;7:275. doi: 10.1038/s41392-022-01096-7 (PMC9381590; doi:10.1038/s41392-022-01096-7)
Supplement: Supplementary file 16 — Supplemental Table 2 [file 41392_2022_1096_MOESM16_ESM.docx]

**Supplemental Table 2.** Univariate and multivariate analysis of factors associated with survival in CRC.

| Characteristics | Univariate analysis | | | Multivariate analysis | | |
| --- | --- | --- | --- | --- | --- | --- |
|  | HR | 95%CI | *p* | HR | 95%CI | *p* |
| DDX39B (low vs. high) | 0.201 | 0.101-0.400 | <0.001 | 0.346 | 0.160-0.747 | 0.007 |
| Age (<60 vs. ≥60) | 0.611 | 0.314-1.190 | 0.147 | 0.854 | 0.413-1.766 | 0.670 |
| Gender (female vs. male) | 0.775 | 0.409-1.468 | 0.435 | 0.683 | 0.339-1.377 | 0.287 |
| Tumor size (<5 cm vs. ≥5 cm) | 0.890 | 0.479-1.653 | 0.712 | 1.718 | 0.819-3.604 | 0.152 |
| Histological grade (well/moderate vs. poor) | 0.289 | 0.156-0.536 | <0.001 | 0.580 | 0.284-1.184 | 0.135 |
| Tumor invasion (T1-T3 vs. T4) | 0.268 | 0.147-0.492 | <0.001 | 0.825 | 0.361-1.882 | 0.647 |
| Lymph node invasion (absent vs. present) | 0.228 | 0.120-0.434 | <0.001 | 0.510 | 0.055-4.737 | 0.554 |
| Distant metastasis (absent vs. present) | 0.092 | 0.036-0.235 | <0.001 | 0.225 | 0.086-0.591 | 0.002 |
| AJCC stage (I-II vs. III-IV) | 0.207 | 0.107-0.399 | <0.001 | 0.370 | 0.176-0.774 | 0.008 |
