# Supplementary material for: DDX39B drives colorectal cancer progression by promoting the stability and nuclear translocation of PKM2
Source: Signal Transduct Target Ther. 2022 Aug 17;7:275. doi: 10.1038/s41392-022-01096-7 (PMC9381590; doi:10.1038/s41392-022-01096-7)
Supplement: Supplementary file 19 — Supplemental Table 5 [file 41392_2022_1096_MOESM19_ESM.docx]

**Supplemental Table 5.** Oligonucleotide sequences used in this study

| **Oligonucleotide name** | | **Sequence (5' to 3')** |
| --- | --- | --- |
| short hairpin RNAs  (shRNAs) | shRNA-negative control (shNC) | TTCTCCGAACGTGTCACGT |
|  | shRNA#1 targeting DDX39B | TAGACATCTCCTCCTACAT |
|  | shRNA#2 targeting DDX39B | CCGCAAGTTCATGCAAGAT |
|  | shRNA targeting PKM2 exon 10 | CATCTACCACTTGCAATTA |
| Primers for qPCR | Human DDX39B forward | TTTGAGCATCCGTCAGAAGTCC |
|  | Human DDX39B reverse | CCAGTACAGACACCTGCCCAGT |
|  | Human PKM2 forward | TGTCTGGAGAAACAGCCAAAGG |
|  | Human PKM2 reverse | CGGAGTTCCTCAAATAATTGCAA |
|  | Human PKM1 forward | TGTCTGGAGAAACAGCCAAAGG |
|  | Human PKM1 reverse | TCCGTCAGAACTATCAAAGCTGCT |
|  | Human GLUT1 forward | CGGGCCAAGAGTGTGCTAAA |
|  | Human GLUT1 reverse | TGACGATACCGGAGCCAATG |
|  | Human LDHA forward | GGCCTGTGCCATCAGTATCT |
|  | Human LDHA reverse | GGAGATCCATCATCTCTCCC |
|  | Huamn Cyclin D1 forward | GTTGCAAAGTCCTGGAGCCT |
|  | Human Cyclin D1 reverse | CACAGGAGCTGGTGTTCC |
|  | Human c-myc forward | ACACCCTTCTCCCTTCG |
|  | Human c-myc reverse | CCGCTCCACATACAGTCC |
|  | Human MEK5 forward | TTCGCATCAAGATCCCAAAT |
|  | Human MEK5 reverse | CAGGCAGAACCTGGCCTAT |
|  | Human STUB1 forward | CAGGGCAATCGTCTGTTCGT |
|  | Human STUB1 reverse | GCTCGTGCTGCTGCATCTT |
|  | Human Sp1 forward | TGCAGCAGAATTGAGTCACC |
|  | Human Sp1 reverse | CACAACATACTGCCCACCAG |
|  | Human ETS1 forward | TGGAGTCAACCCAGCCTATC |
|  | Human ETS1 reverse | TGCAAGGTGTCTGTCTGGAG |
|  | Human c-JUN forward | AGAGCATGACCCTGAACCTG |
|  | Human c-JUN reverse | CCGTTGCTGGACTGGATTAT |
|  | Human β-actin forward | CCTCGCCTTTGCCGATCC |
|  | Human β-actin reverse | GGATCTTCATGAGGTAGTCAGTC |
| Primers for ChIP | Human c-myc promotor forward | CAGCCCGAGACTGTTGC |
|  | Human c-myc promotor reverse | CAGAGCGTGGGATGTTAG |
|  | Human Cyclin D1 promotor forward | GGGGCGATTTGCATTTCTAT |
|  | Human Cyclin D1 promotor reverse | CGGTCGTTGAGGAGGTTGG |
|  | Human MEK5 promotor forward | GTGGGAGAGATTTAATGGTC |
|  | Human MEK5 promotor reverse | GTTACCAGATGTGTTCACCAC |
|  | Human DDX39B promotor Sp1 site 1 forward | GGGGATAGCAAATGTTTCA |
|  | Human DDX39B promotor Sp1 site 1 reverse | TGGGATTACAGGCGTGAG |
|  | Human DDX39B promotor Sp1 site 2 forward | CCGCTCGGATTCAGAACA |
|  | Human DDX39B promotor Sp1 site 2 reverse | CTCAGGGAACTGGATTGCTC |
| Primers for mutant plasmids construction | RNAi-resistant PKM2 mutation forward | AGAGGCTGCCATCTATCATCTACAATTATTTGA |
|  | RNAi-resistant PKM2 mutation reverse | TAGATGATAGATGGCAGCCTCTGCCTCAC |
|  | DDX39B F127A forward | GTCACACTCGGGAGTTGGCTGCTCAGATCAGC |
|  | DDX39B F127A reverse | GCAGCCAACTCCCGAGTGTGACACATCACCAG |
|  | DDX39B L152A forward | TGCTGTTTTTTTTGGTGGTGCGTCTATCAAGAAG |
|  | DDX39B L152A reverse | GCACCACCAAAAAAAACAGCAACCTTGACATTGG |
|  | DDX39B K155A forward | TTTTTTGGTGGTCTGTCTATCGCGAAGGATGAA |
|  | DDX39B K155A reverse | GCGATAGACAGACCACCAAAAAAAACAGCAACC |
|  | DDX39B D206A forward | TAAGATGCTTGAACAGCTCGCCATGCGTCGGG |
|  | DDX39B D206A reverse | GCGAGCTGTTCAAGCATCTTATCACATTCATC |
|  | DDX39B D210A forward | ACAGCTCGACATGCGTCGGGCTGTCCAGGAAATTT |
|  | DDX39B D210A reverse | GCCCGACGCATGTCGAGCTGTTCAAGCATCTT |
|  | DDX39B R319A forward | TCCCAGCCATTGCCATCCACGCTGGGATGCCCCA |
|  | DDX39B R319A reverse | GCGTGGATGGCAATGGCTGGGAAGTTCTGCTCCA |
|  | DDX39B D367A forward | TTAATTATGACATGCCTGAGGCTTCTGACACCTA |
|  | DDX39B D367A reverse | GCCTCAGGCATGTCATAATTAAAAGCAATGTTCA |
|  | DDX39B D369A forward | ATGACATGCCTGAGGATTCTGCCACCTACCTGCA |
|  | DDX39B D369A reverse | GCAGAATCCTCAGGCATGTCATAATTAAAAGC |
|  | PKM2 S37A forward | GCCGCCTGGACATTGATGCACCACCCAT |
|  | PKM2 S37A reverse | CATCAATGTCCAGGCGGCACATGTGCTC |
|  | PKM2 R399/400A forward | TTATTTGAGGAACTCGCCGCCCTGGCGCC |
|  | PKM2 R399/400A reverse | GCGGCGAGTTCCTCAAATAATTGCAAGTGGTAGA |
