# Supplementary material for: DDX39B drives colorectal cancer progression by promoting the stability and nuclear translocation of PKM2
Source: Signal Transduct Target Ther. 2022 Aug 17;7:275. doi: 10.1038/s41392-022-01096-7 (PMC9381590; doi:10.1038/s41392-022-01096-7)
Supplement: Supplementary file 20 — Supplemental Table 6 [file 41392_2022_1096_MOESM20_ESM.docx]

**Supplemental Table 6.** Reagent or resource used in this study

| **REAGENT or RESOURCE** | **SOURCE** | **IDENTIFIER** |
| --- | --- | --- |
| Antibodies | | |
| Mouse monoclonal PKM2 antibody | Sabbiotech | Cat# JM001 |
| Rabbit polyclonal PKM2 antibody | Proteintech Group | Cat# 15822-1-AP |
| Rabbit monoclonal PKM1 antibody | CST | Cat# 7067 |
| Rabbit polyclonal pPKM2 S37 antibody | Sabbiotech | Car# 12822 |
| Mouse monoclonal DDX39B antibody | Sangon Biotech | Cat# D199713 |
| Rabbit polyclonal DDX39B antibody | Proteintech Group | Cat# 14798-1-AP |
| Rabbit polyclonal STUB1 antibody | Sangon Biotech | Cat# D154048 |
| Rabbit monoclonal STAT3 antibody | CST | Cat# 4904 |
| Rabbit polyclonal pSTAT3 Y705 antibody | Sabbiotech | Cat# 11045 |
| Mouse monoclonal Histone H3 antibody | CST | Cat# 14269 |
| Rabbit polyclonal pHistone H3 T11 antibody | Sabbiotech | Cat# 11577 |
| Rabbit monoclonal ERK1/2 antibody | Huabio | Cat# ET1601-29 |
| Rabbit monoclonal pErk1(T202/Y204) +pErk2(T185/Y187) antibody | Huabio | Cat# ET1610-13 |
| Rabbit polyclonal importinα5 antibody | Sangon Biotech | Cat# D154120 |
| Rabbit monoclonal Ki-67 antibody | CST | Cat# 9027 |
| Rabbit polyclonal β-catenin antibody | Huabio | Cat# 0407-16 |
| Mouse monoclonal Lamin B1 antibody | CST | Cat# 17416 |
| Rabbit monoclonal SP1 antibody | CST | Cat# 9389 |
| Rabbit monoclonal c-JUN antibody | CST | Cat# 9165 |
| Rabbit monoclonal ETS1 antibody | CST | Cat# 14069 |
| Rabbit monoclonal ITGA5 antibody | CST | Cat# 98204 |
| Rabbit monoclonal ITGB1 antibody | CST | Cat# 4706 |
| Rabbit monoclonal FAK antibody | CST | Cat# 71433 |
| Rabbit monoclonal pFAK (Y397) antibody | CST | Cat# 8556 |
| Rabbit polyclonal ACTB antibody | Sangon Biotech | Cat# D110001 |
| Rabbit monoclonal Flag tag antibody | CST | Cat# 14793 |
| Mouse monoclonal Flag tag antibody | CST | Cat# 8146 |
| Rabbit Polyclonal HA tag antibody | Proteintech Group | Cat# 51064-2-AP |
| Rabbit monoclonal Myc tag antibody | CST | Cat# 2278 |
| Mouse monoclonal Myc tag antibody | CST | Cat# 2276 |
| Rabbit monoclonal GST tag antibody | CST | Cat# 2625 |
| Rabbit monoclonal His tag antibody | CST | Cat# 12698 |
| Normal rabbit IgG | Sigma-Aldrich | Cat# I5006 |
| HRP-conjugated goat anti-rabbit IgG | Sangon Biotech | Cat# D110058 |
| HRP-conjugated goat anti-mouse IgG | Sangon Biotech | Cat# D110087 |
| Alexa Fluor 488 Goat anti-Rabbit | Invitrogen | Cat# A-11034 |
| Alexa Fluor 594 Goat anti-Rabbit | Invitrogen | Cat# A-11037 |
| Alexa Fluor 488 Goat anti-Mouse | Invitrogen | Cat# A-11029 |
| Alexa Fluor 594 Goat anti-Mouse | Invitrogen | Cat# A-11032 |
| Bacterial | | |
| DH5α | ThermoFisher | Cat# 18265017 |
| BL21 | Transgen | Cat# CD901-02 |
| Biological samples | | |
| Human CRC specimens, normal colorectal tissues, patient-paired liver metastases originating from CRC | West China Biobanks, Department of Clinical Research Management, West China Hospital, Sichuan University | N/A |
| Chemicals, peptides, and recombinant proteins | | |
| Dulbecco’s modified Eagle’s medium | Gibco | Cat# 12800-017 |
| Fetal bovine serum | Gibco | Cat# A3160802 |
| G-418 | Solarbio | Cat# G8160 |
| TRIzon | CoWin Biosciences | Cat# CW0580 |
| SuperScript II Reverse Transcriptase | Invitrogen | Cat# 18064022 |
| SYBR Green qPCR Master Mix | Bimake | Cat# B21203 |
| Glutathione Sepharose 4B | GE Healthcare | Cat# 17-0756-01 |
| Ni-NTA Agarose | Invitrogen | Cat# R901-01 |
| Anti-FLAG M2 Magnetic Beads | Sigma-Aldrich | Cat# M8823 |
| Protein A/G Magnetic Beads | MCE | Cat# HY-K0202 |
| Protease inhibitor cocktail | ThermoFisher | Cat# 78430 |
| Phosphatase Inhibitor | ThermoFisher | Cat# 78428 |
| PVDF membrane | Millipore | Cat# IPVH00010 |
| Chemiluminescent HRP substrate | Millipore | Cat# WBKLS0500 |
| Feto Protein Staining Buffer | Affinibody Life Science AG | Cat# 18.001.10 |
| Matrigel growth-factor reduced | Sigma-Aldrich | Cat# E6909 |
| U0126 | Selleck | Cat# S1102 |
| Cycloheximide | Selleck | Cat# S7418 |
| MG132 | Selleck | Cat# S2619 |
| Transwell Polycarbonate Membrane | Corning | Cat# 3422 |
| Human epidermal growth factor | Sigma-Aldrich | Cat# E5036 |
| 2-NBDG | Sigma-Aldrich | Cat# 72987 |
| DAPI | Solarbio | Cat# C0065 |
| VivoGlo Luciferin | Promega | Cat# P1043 |
| Formic acid | Sigma-Aldrich | Cat# 00940 |
| Acetonitrile | ThermoFisher | Cat# A955-4 |
| 1,4-dithiothreitol | Sigma-Aldrich | Cat# D0632 |
| Iodoacetamide | Sigma-Aldrich | Cat# V900335 |
| Trypsin | Promega | Cat# V5071 |
| ZipTip | Millipore | Cat# ZTC18S096 |
| Critical commercial assays | | |
| Cell-Light EdU Apollo488 kit | RiboBio | Cat# C10310-3 |
| Dual-Luciferase Reporter Assay System | Promega | Cat# E1910 |
| Nuclear and Cytoplasmic Extraction kit | ThermoFisher | Cat# 78833 |
| EZ-Magna ChIP kit | Millipore | Cat# 17-371 |
| Pyruvate Kinase Activity Assay kit | Biovision | Cat# K709 |
| ATP content assay kit | Solarbio | Cat# 0300 |
| Pyruvate content assay kit | Solarbio | Cat# 2205 |
| Lactate content assay kit | Solarbio | Cat# 2235 |
| Cell Counting Kit-8 | Tsbiochem | Cat# C0005 |
| Seahorse XF Glycolysis Stress Test kit | Agilent | Cat# 103020-100 |
| Fast Mutagenesis System kit | TransGen Biotech | Cat# FM111-01 |
| Hematoxylin and Eosin Staining Kit | Beyotime | Cat# C0105M |
| DAB Detection Kit | Gene Tech | Cat# GK600510 |
| Lipofectamine 3000 Transfection kit | Invitrogen | Cat# L3000-015 |
| Deposited data | | |
| Mass spectrometry data of proteins interacted DDX39B | This paper | **Supplemental Table 3** |
| DDX39B and PKM2 crystal structures | Protein Data Bank | PDB ID:1XTI and 1T5A |
| Energy component for the DDX39B-PKM2 complex | This paper | **Supplemental Table 4** |
| Cell lines | | |
| SW620 | ATCC | Cat# CCL-227 |
| HCT116 | ATCC | Cat# CCL-247 |
| SW480 | ATCC | Cat# CCL-228 |
| HT-29 | ATCC | Cat# HTB-38 |
| LoVo | ATCC | Cat# CCL-229 |
| Sublines from SW620 and HCT116 | This paper | N/A |
| Experimental models: organisms/strains | | |
| Mouse: female BALB/c nu (4-6 weeks old) | Gempharmatech Co., Jiangsu | N/A |
| Oligonucleotides | | |
| siRNA #1 for Sp1 | ThermoFisher | Assay ID 116546 |
| siRNA #2 for Sp1 | ThermoFisher | Assay ID 116547 |
| siRNA #1 for ETS1 | ThermoFisher | Assay ID 115623 |
| siRNA #2 for ETS1 | ThermoFisher | Assay ID 115624 |
| siRNA #1 for c-JUN | ThermoFisher | Assay ID 115273 |
| siRNA #2 for c-JUN | ThermoFisher | Assay ID 115274 |
| See **Table S5** for shRNA sequences | This paper | N/A |
| Recombinant DNA | | |
| pCMV-Flag tag vector | kindly provided by Lei Qunyin | N/A |
| pRK7-Myc tag vector | Addgene | Cat# 19400 |
| pcDNA3.1-HA tag vector | Addgene | Cat# 128034 |
| pBiFc-VN173 vector | Addgene | Cat# 22010 |
| pBiFc-VC155 vector | Addgene | Cat# 22011 |
| pET28a-His tag vector | EMD Biosciences | Cat# 69864-3 |
| pGEX-5X-3-GST tag vector | kindly provided by Lei Qunyin | N/A |
| pENTER-Flag-His tag vector | Vigenebio | Cat# PD88001 |
| pRK5-HA-Ubiquitin | Addgene | Cat# 17608 |
| pBiFc-VN173-DDX39B | This paper | N/A |
| pBiFc-VC155-PKM2 | This paper | N/A |
| pENTER-Flag-His-DDX39B | Vigenebio | Cat# CH821820 |
| pENTER-Flag-His-Sp1 | Vigenebio | Cat# CH803234 |
| pENTER-Flag-His-ETS1 | Vigenebio | Cat# CH815030 |
| pENTER-Flag-His-c-JUN | Vigenebio | Cat# CH836318 |
| pCMV-Flag-DDX39B | This paper | N/A |
| pRK7-Myc-DDX39B | This paper | N/A |
| pRK7-Myc-DDX39B R319A | This paper | N/A |
| pCMV-Flag-PKM2 | kindly provided by Lei Qunyin | N/A |
| pcDNA3.1-HA-PKM1 | kindly provided by Lei Qunyin | N/A |
| pGL3-basic | Promega | Cat# E1751 |
| pRL-TK | Promega | Cat# E2241 |
| pGL3-DDX39B promoter (-1500 / +60) | Produced by Vigenebio | N/A |
| pGL3-DDX39B promoter site 1 mutation | Produced by Vigenebio | N/A |
| pGL3-DDX39B promoter site 2 mutation | Produced by Vigenebio | N/A |
| pRK7-Myc-PKM2 | This paper | N/A |
| pRK7-Myc-PKM2 S37A | This paper | N/A |
| pRK7-Myc-PKM2 R399/400A | This paper | N/A |
| pcDNA3.1-HA-PKM2 | This paper | N/A |
| pcDNA3.1-HA-PKM2 1-219aa | This paper | N/A |
| pcDNA3.1-HA-PKM2 1-390aa | This paper | N/A |
| pcDNA3.1-HA-PKM2 219-390aa | This paper | N/A |
| pcDNA3.1-HA-PKM2 219-531aa | This paper | N/A |
| pcDNA3.1-HA-PKM2 390-531aa | This paper | N/A |
| pGEX-5X-3-GST-DDX39B | This paper | N/A |
| pGEX-5X-3-GST-DDX39B F127A | This paper | N/A |
| pGEX-5X-3-GST-DDX39B F152A | This paper | N/A |
| pGEX-5X-3-GST-DDX39B K155A | This paper | N/A |
| pGEX-5X-3-GST-DDX39B D206A | This paper | N/A |
| pGEX-5X-3-GST-DDX39B D210A | This paper | N/A |
| pGEX-5X-3-GST-DDX39B R319A | This paper | N/A |
| pGEX-5X-3-GST-DDX39B D367A | This paper | N/A |
| pGEX-5X-3-GST-DDX39B D369A | This paper | N/A |
| pGEX-5X-3-GST-STUB1 | This paper | N/A |
| pET28a-His-PKM2 | This paper | N/A |
| pGEX-5X-3-GST-importin α5 | Produced by Vigenebio | N/A |
| pGEX-5X-3-GST-β-catenin | Produced by Vigenebio | N/A |
| Software and algorithms | | |
| STRING | Szklarczyk D, *et al*., 2019 | https://www.string-db.org/ |
| GEPIA2 | Tang *et al*., 2019 | http://gepia2.cancer-pku.cn/ |
| UALCAN | Chandrashekar DS *et al.*, 2017 | http://ualcan.path.uab.edu/index.html |
| UCSC Xena | Goldman, M.J. *et al*., 2020 | https://xenabrowser.net/ |
| ZDOCK | Pierce BG, *et al*., | http://zdock.umassmed.edu/ |
| ImageJ | N/A | https://imagej.nih.gov/ij |
| IVIS Living Image Analyze 12.0 | PerkinElmer | N/A |
| GraphPad Prism 8 | GraphPad Prism | https://www.graphpad.com/ scientificsoftware/prism |
